# Emerging Technologies in Blue Foods: Production, Processing, and Omics Perspectives

**DOI:** 10.3390/foods15081390

**Published:** 2026-04-16

**Authors:** Imad Khan, Caimei Wang, Jiangmin Wang, Qiang Zhang, Kunpeng Wang, Ziqian Zhou, Mudassar Hussain, Su Hlaing Phyo, Janice Adaeze Nwankwo, Qiuyu Xia

**Affiliations:** 1 Guangdong Provincial Key Laboratory of Aquatic Product Processing and Safety, Guangdong Province Engineering Laboratory for Marine Biological Products, Guangdong Provincial Engineering Technology Research Center of Seafood, Guangdong Provincial Engineering Technology Research Center of Prefabricated Seafood Processing and Quality Control, College of Food Science and Technology, Guangdong Ocean University, Zhanjiang 524088, China; imadk5577@gmail.com (I.K.); cimy073786@163.com (C.W.); 18719420309@163.com (J.W.); qzzz1274@163.com (Q.Z.); wangkunpeng2077@163.com (K.W.); zhou2022666666@126.com (Z.Z.); 2 Laboratory of Biochemistry and Technology, Lithuanian Research Centre for Agriculture and Forestry, Institute of Horticulture, Kaunas Str. 30, Kaunas District, 54333 Babtai, Lithuania; princemudassar776@gmail.com; 3 State Key Laboratory of Food Science and Resources, National Engineering Research Center for Functional Food, National Engineering Research Center of Cereal Fermentation and Food Biomanufacturing, Collaborative Innovation Center of Food Safety and Quality Control in Jiangsu Province, School of Food Science and Technology, Jiangnan University, 1800 Lihu Road, Wuxi 214122, China; suhlaingphyo.psh22@outlook.com (S.H.P.); jany02413@gmail.com (J.A.N.)

**Keywords:** blue foods, cellular aquaculture, alternative proteins, structuring technologies, non-thermal processing

## Abstract

The growing global population and increasing pressure on conventional food systems have intensified the search for sustainable and nutrient-rich protein sources. Blue foods derived from marine and freshwater organisms offer significant nutritional advantages and lower environmental footprints compared with many terrestrial animal proteins. However, challenges related to resource sustainability, processing, preservation, and product traceability limit their full potential. This review provides a broad overview of emerging technologies shaping the future of blue food systems, covering innovative production strategies, advanced processing techniques, and omics-based analytical approaches. Key developments in cellular aquaculture and cellular mariculture are discussed as promising alternatives to traditional fisheries and aquaculture, enabling the production of blue food through controlled cell cultivation. Additionally, alternative protein platforms including plant-based, fermentation-derived, and cultivated blue food analogues are assessed for their potential to enhance sustainability and diversify aquatic protein sources. Advanced structuring technologies such as extrusion, electrospinning, wet spinning, and 3D printing are highlighted for their roles in developing blue food analogues with improved texture and sensory attributes. Furthermore, non-thermal preservation techniques, including cold plasma (CP), high-pressure processing (HPP), pulsed electric fields (PEFs), and ultraviolet-based treatments, are reviewed for their effectiveness in improving microbial safety and extending shelf life while maintaining nutritional quality. The integration of omics technologies (proteomics, metabolomics, and lipidomics) provides deeper molecular insights into product quality, authenticity, and traceability within blue food supply chains. Collectively, these interdisciplinary advancements demonstrate strong potential to transform blue food production into a more resilient, sustainable, and technology-driven sector. Future progress will depend on overcoming challenges related to scalability, regulatory frameworks, and consumer acceptance to enable the successful commercialization of next-generation blue food products.

## 1. Introduction

Blue foods from marine and freshwater sources represent an important component of sustainable protein supply systems, particularly under increasing global demand. These foods provide high nutritional value with relatively lower environmental impacts compared to terrestrial animal proteins, as they are rich in essential micronutrients, vitamins, and polyunsaturated fatty acids (PUFAs). Although blue foods make significant contributions to society, their role in advancing healthier, more equitable, and environmentally sustainable food systems is frequently underrecognized in policy discussions and academic discourse [1]. When considered, they are usually simplified to just a few types of ‘fish’ in dietary recommendations, and demand projections. Similarly, ocean policies tend to overlook the nutritional benefits and community advantages associated with blue foods [2]. A broad understanding of the diverse functions that blue foods can serve is essential for guiding policy decisions that harness their unique potential to address nutritional, social, and environmental challenges within food systems. Additionally, such knowledge is crucial for effectively managing the trade-offs associated with these roles at both national and international levels.

With the ongoing growth of the global population, the demand for protein has increased substantially, currently estimated at approximately 202 million tonnes for a population of 7.3 billion. Projections indicate that protein demand is expected to rise by an additional 32% to 43% in the coming decades [3]. In coastal and marine-dependent communities, fish and blue food have historically been vital sources of protein. However, recent evaluations by the Food and Agriculture Organization (FAO) of the United Nations highlight ongoing overexploitation in marine capture fisheries, revealing that more than 25% of tracked fish stocks are overfished [4]. Despite the United Nations Sustainable Development Goals targeting the elimination of unreported, illegal, and unregulated (IUU) fishing by 2020, this objective remains unmet. Overfishing continues to be a significant challenge, continued by a complex interplay of factors including corruption, poverty, inadequate regulatory enforcement, excessive fishing capacity, and weak fisheries governance [5]. Although large-scale restoration of marine fisheries remains viable, efforts to rebuild fish populations are increasingly inhibited by rising ocean temperatures and socio-economic pressures, including the necessity to protect critical livelihoods. In response, many countries are expanding aquaculture to enhance protein availability, with particular focus on intensive farming systems that depend on commercially formulated feeds. While this shift has contributed to increased global protein production, it has also worsened environmental concerns, particularly due to the heavy reliance on forage fish in aquafeed production, which may further stress marine ecosystems.

In response to the increasing demand for sustainable protein sources, a variety of innovative alternatives are being investigated to supplement and ultimately reduce dependence on traditional marine capture and aquaculture systems. Insect meal has emerged as a promising feed ingredient, providing essential micronutrients and lipids for the expanding aquaculture sector. Additionally, emerging protein sources such as cell-based mariculture, inshore bivalve farming, plant-derived blue foods, and microalgae cultivation are gaining recognition for their ability to supply high-quality protein with a reduced environmental impact. Strategic investments in these areas have demonstrated potential in mitigating pressure on overexploited fish stocks, including Atlantic cod, various tuna species, and swordfish. Significant investment has been directed toward cell-cultivated blue food technologies, with over $60 million allocated by 2020 [6]. These developments are part of a broader shift toward blue food alternative proteins, encompassing fermentation-derived, plant-based, and cultivated products. Plant-based proteins from grains, legumes, and algae offer significant nutritional benefits while reducing resource consumption compared to conventional meat [7]. Fermentation technologies contribute further by producing single-cell proteins (SCPs) and improving sensory qualities to enhance consumer acceptance. Cultivated meat, although still facing scalability challenges, represents a forward-looking solution with appeal for ethically motivated consumers [8]. Collectively, these approaches respond to the urgent demand for sustainable protein solutions and align with global Sustainable Development Goals, thus enabling the transition toward a more resilient and environmentally sustainable food system.

Innovative biotechnological approaches in blue food production are transforming the industry by integrating advanced techniques to enhance sustainability, nutrition, and sensory qualities. Cellular aquaculture is a growing field which applies tissue engineering to produce blue food without animal slaughter by offering a sustainable alternative to traditional aquaculture. This method involves cultivating cells from marine organisms like fish and mollusks in controlled environments, such as bioreactors, to produce meat-like products with desirable sensory attributes [9]. Additionally, alternative proteins sourced from aquatic organisms, particularly macro- and microalgae, are gaining interest due to their high nutritional content and sustainable cultivation, which does not compete with traditional agriculture for land use [10]. Furthermore, advanced structuring technologies, including extrusion, electrospinning, and 3D printing, are employed to produce textures and structures in food products that mimic traditional blue food by enhancing consumer acceptance [11]. Moreover, non-thermal-preservation methods like high-pressure-processing (HPP), cold plasma (CP), pulsed electric fields (PEFs), and ultraviolet (UV) light are crucial for maintaining the nutritional and sensory qualities of blue foods while ensuring safety and extending shelf life. These methods effectively inactivate microbes without compromising food quality which aligns with sustainability goals [12]. In addition, omics technologies, such as proteomics and metabolomics, are applied to improve the molecular understanding of blue foods, which helps in the enhancement of sensory qualities and safety. These technologies provide insights into the biochemical composition of blue foods by enabling the development of products with improved taste, texture, and nutritional profiles [11]. Despite these promising advancements, challenges such as the scalability of cellular aquaculture and the development of specialized culture media for marine cells remain. Moreover, integrating these technologies into existing food systems requires substantial investment, regulatory oversight, and public acceptance to ensure successful commercialization and consumer trust.

The aim of this review article is to provide a broad overview of emerging technologies driving innovation in blue food systems. It covers advancements in cellular aquaculture and mariculture, as well as alternative protein strategies including fermentation-derived, plant-based and cultivated blue food analogues. The article also discusses advanced structuring technologies and non-thermal-preservation methods that enhance the sensory, safety, and nutritional quality of blue food products. Furthermore, the integration of omics approaches, such as proteomics, metabolomics, and lipidomics, is explored for their potential to optimize product development and ensure traceability. By synthesizing developments across these interdisciplinary domains, this article offers insights to guide research, industry, and policy toward sustainable and resilient aquatic food solutions.

## 2. Emerging Production Technologies in Blue Food Systems

Advancements in innovative production and processing technologies are revolutionizing the blue food sector by offering sustainable alternatives to traditional blue food harvesting. These technologies focus on enhancing protein yield, product quality, and environmental sustainability. Among them cellular agriculture and cellular aquaculture, are gaining attention for their potential to produce aquatic proteins without relying on conventional capture or farming methods.

Furthermore, to ensure conceptual clarity, the terminology used in this review is defined as follows. “Cellular agriculture” refers to the broader production of food products from cultivated animal cells. Within this framework, “cultivated meat” generally describes products derived from terrestrial animal cells, whereas “cultivated blue foods” refers specifically to products obtained from aquatic species such as fish, crustaceans, and mollusks (i.e., seafood). The terms “cellular aquaculture” and “cellular mariculture” denote the application of cell culture technologies to freshwater and marine organisms, respectively. For consistency, this review primarily adopts the term “cultivated blue foods” when referring to cell-based aquatic food products, while the term “seafood” is used to describe the intrinsic structural and compositional characteristics of these organisms. These approaches, along with other emerging techniques, are discussed in detail in the following subsections.

### 2.1. Cellular Aquaculture

A stable blue food supply is essential for maintaining biodiversity and feeding the growing global population. However, this supply is increasingly threatened by overfishing, climate change, and pollution. Blue food includes mollusks, fish, and crustaceans, accounts for approximately 17% of the world’s demand for animal protein [13]. There is expected to be a significant gap between the demand for blue food and its available supply in the near future, considering the present conditions of both aquaculture and wild fisheries. The COVID-19 pandemic has highlighted existing weaknesses in the global food supply chain, emphasizing the critical need for robust and sustainable strategies in animal protein production. Consequently, there is an increasing need for innovative strategies in blue food production aimed at developing efficient, flexible, and resilient systems that can withstand both present and emerging challenges. As noted in the OECD and FAO Agricultural Outlook 2020–2029, aquaculture has maintained a steady annual growth rate of approximately 2%, whereas capture fisheries have declined by 4%, largely attributed to reduced harvests of important fish species [14]. Furthermore, projections indicate that global fish production will exceed 200 million metric tons by 2029, marking a 14% rise from the 2017–2019 average. Despite ongoing growth, the expansion of the sector is projected to slow, with an expected annual growth rate of 1.3%, a decline from the 2.3% recorded in the previous decade. This slowdown is attributed to reduced growth in both fisheries and aquaculture capture. By 2029, it is anticipated that 90% of all fish production will be due for human consumption, marking a 16.3% increase. However, the rate of growth in fish availability for consumption is forecasted to decline from an average annual rise of 2.5% between 2010 and 2019 to just 1.4%. In addition, FAO estimates suggest that by 2050, with the global population projected to reach 9 billion, food production must increase by approximately 70%, and meat production must double to meet the growing dietary needs worldwide [15].

Moreover, the global movement toward climate-resilient food production systems has increased interest in alternative protein sources, particularly cultivated meat, also known as in vitro meat. A significant milestone in this domain occurred in 2013 when Dr. Mark Post introduced the world’s first lab-cultivated hamburger, an achievement that catalyzed international attention and substantial investments in cellular agriculture and alternative protein research [16]. This innovation has shaped new ways for the application of fish-derived muscle cell lines within the context of cellular aquaculture, highlighting the urgent need for the characterization, development, and optimization of such cell lines to enable the sustainable production of fish meat. Furthermore, in vitro models, such as the C2C12 murine myoblast cell line, have significantly advanced our comprehension of the molecular mechanisms supporting myogenesis in mammals. However, analogous research in teleost fish species remains emerging, primarily due to the limited availability of stable and continuous muscle cell lines. While several piscine muscle cell lines have been established, their potential for application in cellular aquaculture has not been extensively investigated.

However, the advancement of cultivated blue foods is depending upon several critical parameters, including the successful isolation and long-term culture of muscle progenitor cells, refinement of culture media and conditions, and the scalability of cell proliferation and differentiation within bioreactor systems. At present, there exists a substantial knowledge gap regarding the genetic and molecular pathways that regulate the differentiation, proliferation, and maturation of fish myogenic precursor cells into fully developed myofibers and myofibrils. Addressing this gap is imperative for progress in cultivated fish meat technologies and requires comprehensive investigation into the biochemical and genetic regulation of muscle development in aquatic species.

Despite the rapid progress in cellular aquaculture, several critical challenges remain that limit its translation from laboratory research to industrial-scale applications. One of the primary bottlenecks is the lack of well-characterized, stable fish muscle cell lines, which constrain reproducibility and scalability compared to mammalian systems. Additionally, the development of cost-effective, serum-free culture media tailored to marine species remains a significant hurdle, particularly given the unique osmotic and metabolic requirements of aquatic cells. Bioreactor design and scale-up strategies also require further optimization to accommodate the distinct growth kinetics and structural requirements of fish cells. Moreover, inconsistencies in reported growth performance and differentiation efficiency across species highlight the need for standardized protocols and deeper mechanistic understanding. Addressing these limitations, alongside regulatory and economic considerations, will be essential to realize the commercial potential of cellular aquaculture and to position it as a viable contributor to sustainable blue food systems.

### 2.2. Cellular Mariculture

Cellular mariculture also referred to as cell-based blue food, involves the in vitro cultivation of cells derived from marine organisms such as shellfish and fish, with the goal of producing edible blue food products. This technique involves isolating cells from marine species and growing them under controlled laboratory conditions, followed by harvesting and processing to produce consumable food items. At present, the production of cellular mariculture is primarily limited to laboratory settings, and large-scale commercial availability has yet to be achieved.

Although the broader cultivated blue foods sector has predominantly focused on terrestrial animal products, recent advancements are being made to extend this technology to marine organisms. These include species such as yellowtail amberjack, bluefin tuna, crab, shrimp, sea cucumber, whitefish, Atlantic salmon, trout, and Coho salmon. A number of pioneering companies such as BlueNalu, Shiok Meats, Finless Foods, Another Fish, Avant Meats, Cell Ag Tech, Bluu Seafood, and Wildtype are actively working on the development and scaling of cellular mariculture technologies. For instance, BlueNalu has formed strategic collaborations with international companies including Thai Union Group (Thailand), Pulmuone (South Korea), and Mitsubishi Corporation (Japan) to accelerate the commercialization of cell-cultivated blue food products [17]. Despite such progress, the availability of these products to consumers remains minimal. As of now, the only cell-cultivated meat product approved for sale is a chicken-based item by Eat Just a San Francisco-based company, which is currently available exclusively in Singapore [18]. Nonetheless, increasing interest and significant financial investment are driving the advancement of cultivated blue food technologies. A notable example is the $100 million Series B funding round secured in 2022, supported by prominent investors and firms associated with high-profile individuals such as Leonardo DiCaprio, Jeff Bezos, agribusiness leader Cargill, and Robert Downey Jr [19].

Furthermore, as of late 2022, no cellular mariculture products had achieved commercial viability, with most efforts remaining focused on research and development. For instance, Shiok Meats announced in 2020 its intention to launch the first cell-cultivated shrimp. However, by 2022, the company was still in the development stage, operating research facilities across Australia, Singapore, Thailand, and Vietnam [19]. Similarly, other companies in the cellular blue food sector projected the commercial availability of their products in retail markets by 2025. During 2022, several of these firms actively sought regulatory approval, particularly in progressive jurisdictions such as Singapore [20]. Although cellular mariculture shares many of the scientific and technological foundations of the broader cultivated meat industry, it has attracted distinct investor interest due to its strong economic potential. Forecasts indicate that the clean blue food market could experience substantial growth, with projected profits reaching billions of dollars, driven by rising consumer demand for sustainable and ethically produced blue food alternatives [14].

Even with growing investment and technological progress, cellular mariculture remains at an early developmental stage, with several critical barriers limiting its commercial realization. A major challenge lies in the complexity of marine cell biology, including species-specific requirements for salinity, temperature, and nutrient composition, which complicate the development of standardized culture systems. In addition, the high cost and scalability limitations of culture media and bioreactor systems continue to hinder industrial translation. While significant financial investments and industry partnerships indicate strong market potential, there is still a lack of publicly available data on production efficiency, product quality, and long-term safety, raising concerns about transparency and reproducibility. Furthermore, regulatory frameworks for cultivated blue food products remain underdeveloped and vary across regions, potentially delaying market entry. Addressing these scientific, technical, and regulatory challenges will be essential to transition cellular mariculture from a promising concept to a viable and competitive component of future blue food systems.

### 2.3. Alternative Protein Strategies for Blue Food System

In the context of blue food systems, the emergence of alternative proteins is driven by sustainability challenges associated with overfishing, aquaculture intensification, and marine ecosystem degradation. These innovative approaches seek to deliver adequate and nutritious food while employing more resource-efficient and environmentally sustainable methods. In recent years, the alternative protein sector has experienced notable growth, propelled by advancements in scientific research, increased commercial investment, and the establishment of supportive regulatory frameworks. However, despite the considerable promise and well-documented benefits of these technologies, a variety of challenges and uncertainties continue to hinder their widespread adoption. Therefore, this section provides a broad overview of the current overview of alternative proteins, with a particular focus on the evolving domain of blue food analogue.

Moreover, alternative proteins include non-animal-based substitutes derived from sources such as microbial fermentation processes, plants, and cultivated animal cells grown in vitro. These approaches aim to replicate the sensory and functional characteristics of conventional blue food products associated with intensive livestock production. Similarly, the concept of blue foods analogue refers to blue food analogues produced using fermentation-derived compounds, plant-based ingredients, or cellular agriculture technologies. These products are engineered to match the texture, taste, appearance, and nutritional properties of traditional aquatic foods. These systems enable controlled production processes with reduced biological variability, thus enabling greater control, precision, and resource efficiency throughout the production process [21], including suboptimal feed conversion rates, metabolic energy losses, susceptibility to disease, and physical injury. Moreover, the shift towards alternative protein and blue food systems offers substantial benefits in terms of food safety, animal welfare, and public health by minimizing reliance on antibiotics, reducing the risk of zoonotic disease transmission, and eliminating the challenges related to the handling and processing of live animals [22].

Furthermore, alternative proteins can be developed through a range of approaches, depending on their source and production philosophy. While some focus exclusively on vegan formulations that exclude all animal-derived ingredients, others explore more inclusive alternatives such as low-trophic level marine organisms, edible insects, underutilized aquatic and terrestrial species, and food industry by-products. The development of alternative proteins can be broadly classified into three key stages: (1) the selection and use of non-conventional raw materials, (2) the application of novel production technologies and processes, and (3) the formulation of diverse protein-rich products tailored to various consumer needs.

At present, the alternative protein sector is primarily driven by three core technological platforms: plant-based systems, microbial fermentation, and cellular agriculture. Plant-based systems rely on crops such as legumes, grains, and other botanical sources to develop protein-rich food products that emulate the sensory and functional properties of animal-derived foods. Microbial fermentation harnesses the biosynthetic capabilities of microorganisms to produce proteins or enhance the nutritional and functional properties of food ingredients. Meanwhile, cellular agriculture involves the in vitro cultivation of animal cells under controlled conditions to produce cultivated meat and blue food analogues. Each of these platforms presents distinct benefits and limitations with respect to environmental sustainability, production scalability, nutritional adequacy, and consumer acceptance, all of which influence their potential for widespread adoption in global food systems.

Even though the rapid expansion of alternative protein technologies, their application to blue food systems remains constrained by several critical limitations. One key challenge is the difficulty in accurately replicating the complex sensory attributes of aquatic foods, particularly the unique texture, flavor, and lipid composition associated with marine species. While plant-based and fermentation-derived approaches offer scalability and sustainability advantages, they often fall short in delivering equivalent nutritional profiles, especially with respect to long-chain omega-3 fatty acids. In contrast, cellular agriculture provides closer biological fidelity but is hindered by high production costs, technical complexity, and limited scalability. Furthermore, discrepancies in life cycle assessment outcomes and environmental impact claims highlight the need for more standardized and transparent evaluation frameworks. Consumer acceptance also remains uncertain, particularly for highly engineered or unfamiliar products. Therefore, future research should prioritize improving sensory realism, nutritional equivalence, and process efficiency, while also addressing regulatory, economic, and consumer perception challenges to enable broader adoption of blue food analogue proteins.

#### 2.3.1. Plant-Based Proteins

Plant-based proteins represent a key component of alternative protein strategies within blue food systems, utilizing plant-derived ingredients to replicate the nutritional, sensory, and structural characteristics of traditional seafood products such as fish and shellfish. These alternatives are specifically formulated to mimic the texture, flavor, appearance, and nutrient composition of blue foods. These systems are designed to replicate blue food characteristics using plant and algal ingredients [21]. The production of plant-based blue food analogues begins with sourcing raw materials such as cereals, legumes, fungi, and marine biomass. These materials undergo fractionation to isolate key components, including proteins, lipids, carbohydrates, and dietary fiber. Subsequent processing employs advanced technologies such as shear cell processing, high-moisture extrusion, and 3D food printing, which restructure these components into functional ingredients most notably textured plant proteins. These are then formulated and combined to produce end products that closely mimic the organoleptic and nutritional characteristics of traditional animal-derived foods, including mouthfeel, flavor, aroma, and appearance. Currently, many commercially available plant-based products are focused on replicating relatively simple food formats such as ground meat, burgers, sausages, and dairy substitutes.

**Figure 1:**
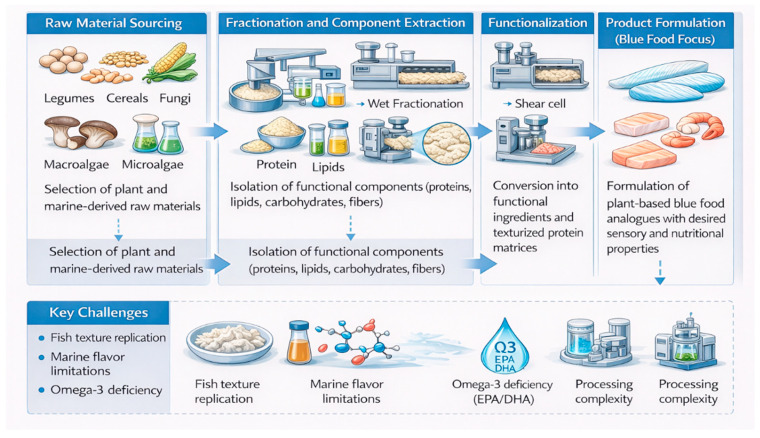
Value chain tailored to technologies used in plant-based product development.

In addition, the process of plant-based protein production, as illustrated in Figure 1, comprises four main stages. In the first stage, raw materials such as crops, mushrooms, and seaweed are cultivated and sourced. Following this, during the production phase, these raw materials are subjected to fractionation processes to extract key components, including proteins, oils, carbohydrates, and fibers. The third stage involves the conversion of these components into functional ingredients through various processing techniques, such as texturization. Finally, in the fourth stage, these functional ingredients are formulated into final products that exhibit specific desired attributes, including taste, aroma, texture, appearance, and nutritional value.

Although significant advancements in plant-based protein technologies, replicating the complex structural and sensory properties of blue foods remains a major challenge. Unlike terrestrial meat, aquatic muscle tissues exhibit unique flake-like structures and delicate textures that are difficult to reproduce using current plant protein matrices. Additionally, plant-based analogues often lack key nutritional components inherent to marine foods, particularly long-chain omega-3 fatty acids, which may require fortification strategies that increase formulation complexity. The reliance on extensive processing and additives to achieve desirable sensory attributes also raises concerns regarding product “clean label” perception and consumer acceptance. Furthermore, variability in raw material quality and functionality can impact product consistency and scalability. Therefore, future efforts should focus on improving structure–function relationships in plant protein systems, enhancing nutritional equivalence, and developing more efficient and sustainable processing strategies tailored specifically to blue food analogues.

#### 2.3.2. Fermentation-Based Protein Production

Fermentation-based protein production has emerged as a key technological platform within blue food systems, enabling the development of sustainable blue food alternatives and functional ingredients with enhanced nutritional and sensory properties. Unlike traditional fermentation primarily associated with conventional food products, modern fermentation approaches in blue food applications focus on the biosynthesis of microbial biomass, marine-derived proteins, and high-value metabolites that can replicate the compositional and functional attributes of blue food. Microorganisms such as microalgae, bacteria, and fungi are increasingly utilized due to their ability to efficiently convert substrates into proteins, lipids, and bioactive compounds relevant to aquatic food systems [11,23].

Among these approaches, biomass fermentation plays a crucial role in producing SCPs derived from marine or marine-adapted microorganisms, offering a sustainable protein source with high nutritional value and reduced environmental impact. Microalgae, in particular, have gained significant attention in blue food innovation due to their capacity to produce long-chain omega-3 PUFAs, such as eicosapentaenoic acid (EPA) and docosahexaenoic acid (DHA), which are essential components of blue food nutritional profiles [11,24]. In addition, precision fermentation enables the targeted production of functional ingredients, including marine-like flavor compounds, pigments, and binding agents, which contribute to improving the sensory quality and consumer acceptance of plant-based and hybrid blue food analogues [11,23]. These capabilities position fermentation as a critical tool for enhancing both the nutritional equivalence and functional performance of blue food analogue products.

**Figure 2:**
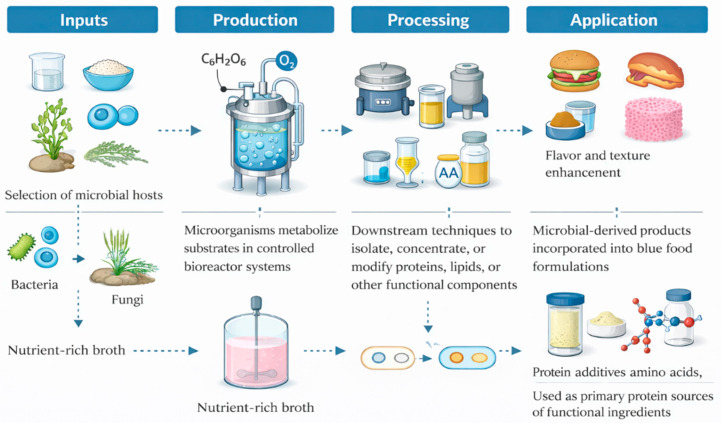
The fermentation pathway: From raw materials to final products and their uses.

The fermentation-based production process, as illustrated in Figure 2, can be divided into four key stages. The initial “Inputs” stage involves the selection of microbial hosts, including algae, bacteria, or fungi, along with suitable feedstocks such as simple sugars or marine biomass. During the “Production” phase, these microorganisms metabolize the substrates under controlled conditions, typically in bioreactor systems, to generate biomass or targeted metabolites. This is followed by the “Processing” stage, where downstream techniques are applied to isolate, concentrate, or modify proteins, lipids, or other functional components. Finally, in the “Application” stage, these microbial-derived products are incorporated into blue food formulations, either as primary protein sources or as functional ingredients that enhance texture, flavor, and nutritional quality.

Although its significant potential, fermentation-based blue food production faces several challenges that limit its large-scale implementation. Key constraints include high production and downstream processing costs, scalability limitations associated with industrial bioreactors, and regulatory uncertainties for novel microbial-derived ingredients. In addition, accurately replicating the complex sensory characteristics of blue foods, particularly marine-specific flavor profiles and omega-3-rich lipid compositions, remains a major challenge. The reliance on refined substrates such as sugars further raises sustainability concerns unless alternative feedstocks from waste streams or circular bioeconomy approaches are adopted. Variability in microbial performance and limited consumer familiarity with fermentation-derived products also hinder broader adoption. Addressing these limitations will require advances in strain engineering, development of cost-effective and sustainable substrates, and improved strategies to enhance sensory and nutritional fidelity. Table 1 provides a comparative overview of emerging production technologies in blue food systems, highlighting their core processes, advantages, limitations, and current industry developments.

#### 2.3.3. Seafood-Specific Structural and Compositional Challenges

A major challenge in the development of blue food analogues is the need to reproduce seafood-specific structural, biochemical, and sensory attributes rather than only general protein functionality. Fish muscle exhibits a unique composition rich in proteins, lipids, and water, with long-chain omega-3 polyunsaturated fatty acids such as EPA and DHA being key nutritional components [25,26]. These lipids are highly susceptible to oxidative degradation, leading to the formation of volatile compounds that significantly influence both nutritional quality and sensory characteristics during processing and storage [27,28,29,30,31]. Furthermore, seafood flavor is driven by complex interactions among lipid oxidation products, amino acids, and other metabolites, which generate characteristic marine aroma compounds that are difficult to replicate in alternative protein systems [27]. In addition, the compositional variability of fish lipids across species further complicates the design of consistent blue food analogues [32]. These factors collectively highlight the intrinsic complexity of seafood systems and the challenges faced in replicating their structure, nutritional composition, and sensory properties.

Furthermore, regardless of advances in alternative protein technologies, current approaches remain limited in their ability to simultaneously address structural organization, lipid stability, and authentic marine flavor generation. While plant-based and extrusion-based systems can produce bulk fibrous textures, they often fail to capture the delicate flake-like behavior and compositional heterogeneity associated with fish muscle. Similarly, although fermentation-based systems enable the production of omega-3-rich lipids, maintaining their stability within food matrices remains challenging due to their high susceptibility to oxidation. In addition, flavor replication strategies frequently rely on external additives rather than intrinsic biochemical pathways, resulting in products that lack the depth and complexity of natural seafood. These limitations indicate that existing technologies are largely fragmented, addressing individual attributes rather than the integrated functionality of blue foods. Therefore, future research should focus on multi-scale structuring approaches, improved lipid stabilization strategies, and integrated flavor generation systems to better replicate the unique characteristics of seafood.

**Table 1:** Comparative overview of emerging production technologies in blue food systems, including cellular aquaculture, cellular mariculture, and alternative protein strategies.

Technology Category	Core Process/Steps	Technology Readiness Level (TRL) *	Scalability	CAPEX/OPEX	Product Fidelity	Sensory Realism	Nutritional Equivalence	Advantages	Challenges/Limitations	Examples/Companies	Industrial & Commercial Status	References
Cellular (Cultivated) Blue Food Systems	Isolation of cells from aquatic species (fish, crustaceans, mollusks); expansion in bioreactors under controlled conditions; differentiation using scaffolds to develop structured blue food products	Low-Medium	Low (currently pilot scale)	Very high	Very high (biological equivalence)	High (muscle-like structure possible)	Very high (can match native composition)	Closest biological and nutritional equivalence to conventional blue food; reduced reliance on wild fisheries; improved food safety and pathogen control; customizable nutritional profiles	High production and media costs; complex cell line development (especially marine species); scalability challenges; regulatory uncertainty; limited consumer acceptance	BlueNalu (finfish), Finless Foods (tuna), Wildtype (salmon), Shiok Meats (shrimp), Avant Meats (fish products)	Early stage commercialization; limited regulatory approvals (e.g., case-by-case approvals in selected regions); no large-scale market presence; high cost remains a major barrier to commercialization	[19,23,33,34,35,36]
Plant-Based Blue Food Analogues	Extraction and processing of plant and algal proteins; fractionation and formulation; structuring using extrusion and related technologies to mimic blue food texture and appearance	High	High (commercial scale)	Low-Medium	Moderate	Moderate (limited replication of flake-like fish texture)	Moderate (requires omega-3 fortification)	Sustainable and resource-efficient; scalable production; established processing technologies; lower cost compared to cellular systems; reduced environmental footprint	Difficulty replicating marine flavor and texture; lower omega-3 content; processing complexity; sensory limitations; consumer acceptance challenges	Good Catch (plant-based tuna), Ocean Hugger Foods (plant-based fish), Sophie’s Kitchen (vegan blue food products), Gathered Foods	Fully commercialized with global market presence; widely approved under existing food regulations; diverse product portfolio available; competitive pricing compared to conventional blue food	[11,37,38]
Fermentation-Based Blue Food Systems	Biomass and precision fermentation using microorganisms (microalgae, bacteria, fungi); production of proteins, lipids (EPA/DHA), and functional ingredients; downstream processing and incorporation into blue food products	Medium	Medium-High	Medium-High	Low-Moderate (mainly ingredient-based)	Moderate (flavor enhancement possible)	High (customizable, e.g., omega-3 production)	High scalability potential; production of omega-3-rich lipids; consistent product quality; reduced resource requirements; potential for circular bioeconomy integration	High production and downstream costs; challenges in replicating marine flavor; regulatory barriers; dependence on feedstock sustainability; limited consumer familiarity	Perfect Day (precision fermentation), The EVERY Company (protein fermentation), marine fermentation-based ingredient developers	Emerging commercialization primarily at ingredient level; regulatory approvals evolving; increasing industrial investment; moderate market penetration with ongoing scale-up efforts.	[23,24,36]

* Abbreviations: TRL, Technology Readiness Level; CAPEX, Capital Expenditure; OPEX, Operational Expenditure; EPA, Eicosapentaenoic Acid; DHA, Docosahexaenoic Acid.

## 3. Structuring Technologies for Blue Food Analogues

Structuring technologies are essential for reproducing the hierarchical organization of blue foods, particularly fibrous and flake-like muscle structures. Several advanced techniques such as electrospinning, extrusion, wet spinning and 3D printing have been explored for their applicability in the development of blue food analogues [39]. A critical aspect of this development involves replicating the appropriate elasticity and textural attributes that are perceived during mastication. While extrusion remains a widely adopted method, particularly in the production of plant-based meat analogues, it can be cost-prohibitive due to high processing expenses. To mitigate these limitations, alternative structuring approaches have been proposed. One such strategy involves the incorporation of plant-derived proteins with polysaccharides, such as alginates, and cross-linking enzymes like microbial transglutaminase, to form robust gel matrices with desirable structural integrity [40]. A detailed discussion of these and other texturization methods relevant to blue food alternatives is provided in the subsequent sections.

### 3.1. Extrusion

Extrusion is a key processing technique used in the development of alternative protein products, including plant-based blue food analogues. It functions by making a mixture of raw ingredients through a die under controlled temperature and pressure conditions, resulting in a textured and shaped product. This method is generally classified into two categories based on moisture content: high-moisture extrusion (40–80% moisture) and low-moisture extrusion (10–35% moisture) [41]. Its widespread adoption in the food industry is attributed to its operational flexibility, cost-effectiveness, scalability, and ability to produce consistent, high-quality outputs. Extruders are typically distinguished by their screw configuration, with twin-screw and single-screw designs being most common. Twin-screw extruders are preferred for producing dense, meat-like textures due to their enhanced shearing and mixing capabilities [42]. A significant benefit of extrusion cooking is the removal of undesirable flavors and odors, such as bitterness, which can enhance the sensory appeal of the final product. Low-moisture extrusion is primarily used to produce meat alternatives that do not require a fibrous structure, such as snack-type products. In contrast, high-moisture extrusion often incorporating a cooling die enables the production of meat-like textures, fibrous, closely mimicking the structural qualities of traditional animal meat [43]. Beyond structural development, extrusion denatures plant proteins, improving their digestibility and nutritional properties while eliminating anti-nutritional compounds. The overall process involves feeding raw plant materials into the extruder, followed by stages of shaping, cooking, cooling, drying, cutting, and packaging to yield the final consumer-ready product.

Although its industrial maturity, extrusion technology faces notable limitations in replicating the delicate and heterogeneous structures characteristic of blue foods. While high-moisture extrusion can generate fibrous textures resembling muscle tissue, it often fails to reproduce the layered, flaky architecture typical of fish and blue food products. Additionally, the high temperature and shear conditions involved may lead to degradation of heat-sensitive nutrients and bioactive compounds, potentially reducing nutritional quality. The process also requires precise control of formulation and operating parameters, as small variations can significantly affect product texture and consistency. Furthermore, extrusion alone is often insufficient to achieve full sensory equivalence, necessitating integration with other structuring or flavor-enhancement strategies. Therefore, future advancements should focus on optimizing process conditions, preserving nutritional integrity, and combining extrusion with complementary technologies to better mimic the unique properties of blue food products.

### 3.2. Electrospinning

Electrospinning is an economical and highly effective technique for fabricating fibers at the nano and micro scale, typically ranging from approximately 100 nanometers to a few micrometers in diameter. The method is based on electrohydrodynamic principles and is capable of producing micro-, submicro-, and nanofibers with high precision. A variety of materials can be employed in this process, including naturally derived substances such as polysaccharides and proteins, as well as synthetic polymers like polycaprolactone (PCL) and polyvinyl alcohol (PVA) [44]. Electrospinning is a technique that employs a high-voltage electric field applied between a polymer solution droplet at the tip of a spinneret and a grounded collector. When the electrostatic forces exceed the surface tension of the droplet, a charged jet is emitted from the polymer solution. This jet experiences elongation and whipping instabilities due to repulsion among like-charged elements. As the solvent evaporates during the jet’s trajectory, continuous fibers are formed and accumulate on the collector, producing a nonwoven mat composed of nanofibers.

Though its ability to produce highly controlled micro- and nano-scale fibrous structures, the application of electrospinning in blue food systems remains largely at the experimental stage. One of the primary limitations is its low production throughput, which restricts scalability for industrial food manufacturing. Additionally, the use of high-voltage systems and, in many cases, organic solvents raises safety, regulatory, and food-grade compatibility concerns that must be carefully addressed before commercialization. While electrospinning shows promise in mimicking fine muscle fiber architectures, translating these structures into bulk, three-dimensional food products with desirable texture and mouthfeel remains challenging. Furthermore, the integration of electrospun fibers into complex food matrices without compromising structural integrity or sensory quality is still not well established. Therefore, future research should focus on developing solvent-free or food-grade systems, improving scalability, and integrating electrospinning with other structuring techniques to enhance its practical applicability in blue food analogue development.

### 3.3. 3-D Printing

3D printing is a form of additive manufacturing, has emerged as a promising technology in the food industry, particularly for the development of customized meat analogues derived from meat by-products. Utilizing digital fabrication and computer-aided design (CAD) software systems, this method allows for the precise construction of 3D food structures without the need for traditional tools or molds [45]. Often referred to as bioprinting when applied to food, the process involves the extrusion of a plant protein-based paste, often combined with polysaccharides, fats, and water, through a nozzle to build layered constructs that replicate the structure and texture of conventional meat products [46]. Materials suitable for 3D food printing extend beyond biological substrates to include ceramics, polymers, and metals in other applications. In the context of alternative proteins, 3D printing offers considerable flexibility in tailoring nutritional composition, sensory attributes, and texture to meet the needs of specific consumer demographics [47]. Furthermore, the technique supports sustainability objectives by reducing energy consumption, raw material usage, transportation, labor, and demands. For successful implementation in food design, careful calibration of printing parameters is essential to achieve the desired structural integrity and appearance. In particular, producing delicate, fibrous textures analogous to fish tissue requires the use of nozzles with smaller diameters. However, maintaining optimal product quality at high printing speeds and minimal nozzle heights presents technical challenges related to precision, material flow, and mechanical stability of the final construct.

Nevertheless, despite its high design flexibility, 3D printing of blue food analogues faces several practical and technological limitations. One of the primary challenges is the restricted range of printable food materials, as suitable formulations must exhibit precise rheological properties to ensure stable extrusion and structural integrity. Achieving the fine, anisotropic structures characteristic of fish muscle remains difficult, particularly at higher printing speeds where resolution and accuracy are compromised. Additionally, the relatively slow production rate compared to conventional processing methods limits its scalability for large-scale food manufacturing. Post-processing steps are often required to enhance texture and stability, adding complexity to the production workflow. Furthermore, the high cost of specialized equipment and the need for precise process control may hinder widespread industrial adoption. Therefore, future research should focus on improving printable material formulations, enhancing printing speed and resolution, and integrating 3D printing with complementary structuring technologies to better replicate the unique properties of blue food products.

### 3.4. Wet-Spinning

**Figure 3:**
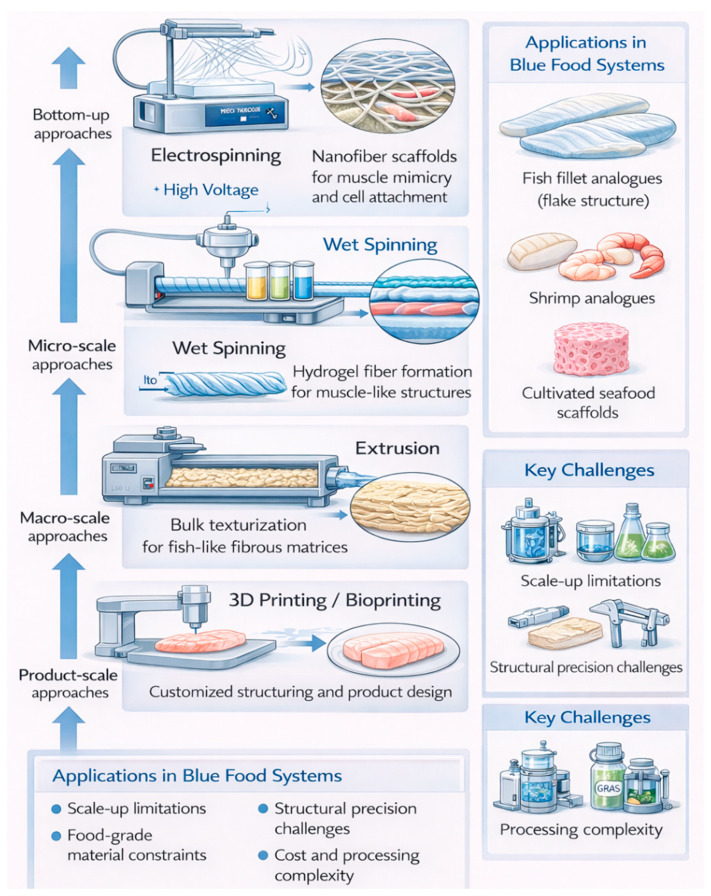
Structuring technologies in alternative protein production: extrusion, electrospinning, wet spinning, and 3D printing/bioprinting for texture, functionality, and customization.

Wet spinning is a fiber fabrication technique used to produce high-quality protein-based fibers, relying on chemical agents, purified proteins, acidic conditions, and elevated salt concentrations. Similar in principle to industrial extrusion methods, wet spinning offers the advantage of modifying the spinning environment to optimize fiber formation. Sodium alginate is widely recognized as a valuable functional compound in the food sector due to its ability to rapidly cross-link with calcium chloride, making it particularly suitable for producing hydrogel fibers through wet spinning. Despite its potential, the broader application of wet-spun fibers in food systems has been limited by the frequent use of synthetic polymers, organic solvents, and chemical cross-linkers, which raise safety and sustainability concerns. To expand the usability of this technique within food contexts, researchers have emphasized the importance of adopting more environmentally friendly solvents and reactants [48]. Food-grade substances, including stabilizers, flavor enhancers, aromatic compounds and colorants have been identified as suitable alternatives. Moreover, combining natural proteins such as zein (from maize) or gluten (derived from wheat) with biopolymers has shown potential in enhancing the functional performance of wet spinning, particularly in the development of textured meat analogues [49]. Figure 3 presents a multi-scale overview of structuring technologies applied in the development of blue food analogues, highlighting how extrusion, electrospinning, wet spinning, and 3D printing operate across nano-, micro-, and macro-structural levels. Unlike conventional representations, this framework emphasizes the complementary roles of these technologies in replicating the hierarchical organization of aquatic muscle, particularly the flake-like and fibrous structures characteristic of fish tissue. It also integrates key application pathways and technological limitations, providing a more comparative and mechanistic understanding of how structuring approaches contribute to texture, functionality, and product design in blue food systems. Table 2 provides a comparative overview of emerging structuring technologies applied in the development of blue food analogues. It highlights their fundamental processing principles, potential applications, and advantages, while also outlining key technical and scalability challenges. Together, these insights illustrate how each technology contributes uniquely to advancing texture, functionality, and sustainability in blue food alternatives.

Even with its potential for producing well-aligned protein fibers, wet spinning faces several challenges that limit its broader application in blue food analogue development. One major limitation is the reliance on chemical coagulation baths and cross-linking agents, which may introduce safety, environmental, and regulatory concerns if not carefully controlled. Additionally, the process often requires multiple downstream steps, including washing and stabilization, which increase complexity and production costs. While wet spinning can generate fibrous structures, it remains difficult to achieve the multi-scale organization and mechanical properties characteristic of natural fish muscle. Furthermore, the scalability of wet spinning for food-grade applications has not yet been fully demonstrated at an industrial level. Therefore, future research should focus on developing greener processing systems, improving structural complexity, and integrating wet spinning with other structuring technologies to enhance its applicability in sustainable blue food production.

## 4. Non-Thermal and Emerging Processing Technologies for Blue Foods

Food quality is influenced by both measurable attributes such as structural integrity and chemical composition and subjective factors, including consumer perceptions. Traditionally, the preservation of fresh and highly perishable foods has mainly depended on thermal processing techniques, with sterilization serving as a key method. Over the last twenty years, conventional thermal treatments like sterilization, pasteurization, and high-temperature drying have continued to dominate food preservation strategies. Although these techniques are effective in prolonging shelf life, they frequently lead to adverse effects on sensory and nutritional attributes, causing undesirable changes in flavor, texture, color, and nutritional composition as a result of extended exposure to high temperatures [62].

To address the limitations of traditional thermal preservation methods, increasing focus has been placed on non-thermal technologies. Innovative techniques such as high-intensity ultrasound, pulsed light, high-pressure-processing, oscillating magnetic fields, ionizing radiation, PEFs, CP and UV light have shown promising potential in enhancing the shelf life of food products while better preserving their nutritional value and sensory characteristics [62]. A critical concern in the preservation of blue foods, particularly due to their common consumption in raw or minimally processed forms, is the assurance of microbiological safety on a global scale. Foodborne pathogens including *Listeria monocytogenes*, Norovirus, *Salmonella* spp., *Campylobacter* spp., *Staphylococcus aureus*, and *Clostridium perfringens* continue to be major causes of illness and mortality and are frequently identified in both blue food commodities and their processing environments. For example, a recent case in China associated the presence of COVID-19 with frozen white shrimp imported from Ecuador, highlighting the urgent need for advanced preservation strategies and safe food additives that can ensure microbiological integrity while maintaining desirable sensory attributes.

### 4.1. Cold Plasma (CP) Technology

Non-thermal preservation technologies provide an effective strategy for microbial inactivation in foods without the application of high temperatures, thus maintaining both safety and extended shelf life while preserving the sensory and nutritional qualities of the product. These technologies offer a balanced approach by integrating food safety with cost-efficiency, minimal processing, and the preservation of product quality through innovative techniques that complement traditional methods. Among the newly developed preservation methods, CP has garnered considerable interest due to its versatile applications within the food industry.

Moreover, CP is produced when gases whether singular or in mixtures are exposed to electric fields of sufficient strength to exceed their ionization threshold, resulting in the formation of plasma. This ionized state comprises a complex mixture of reactive components such as free radicals, positive and negative ions, electrons, excited and neutral molecules, and electromagnetic radiation including visible light and UV [63]. CP has demonstrated versatility not only in food processing but also in biomedical applications, such as wound healing, antimicrobial treatment, and cancer therapy. Furthermore, in the context of food preservation, CP has been employed to extend the shelf life of perishable products like shrimp, fish, meat, and vegetables and fresh fruits [64]. The effectiveness of CP in microbial decontamination is mainly attributed to the production of reactive oxygen species (ROS) and reactive nitrogen species (RNS), including hydrogen peroxide, ozone, nitrogen oxides, and singlet oxygen which possess potent antimicrobial properties [64]. However, while its bactericidal capabilities are well-documented, further investigation is needed to understand the broader effects of CP on the physicochemical and sensory attributes of treated food products.

Although its strong antimicrobial potential, the application of CP in blue food systems presents several limitations that require further investigation. One of the primary concerns is the potential for oxidative damage induced by reactive species, which may adversely affect lipids, proteins, and pigments, particularly in PUFA-rich aquatic products. This can lead to quality deterioration, including off-flavors, discoloration, and reduced nutritional value. Additionally, the lack of standardized processing parameters across studies makes it difficult to compare results and optimize treatment conditions for different blue food matrices. The penetration depth of plasma treatment is also limited, potentially reducing its effectiveness in thicker or complex food structures. Furthermore, industrial scalability and integration into existing processing lines remain challenging due to equipment design and process control requirements. Therefore, future research should focus on balancing antimicrobial efficacy with quality preservation, establishing standardized protocols, and improving system design for practical application in blue food preservation.

#### Preservation of Blue Food by CP

Blue foods including a diverse group of aquatic organisms such as mollusks, fish, echinoderms, and crustaceans are highly valued for their exceptional nutritional value, offering abundant sources of essential fatty acids, proteins, minerals and vitamins. These marine-derived products are widely valued not only for their health benefits but also for their favorable sensory attributes. Nonetheless, their post-harvest shelf life is notably limited due to intrinsic characteristics, including neutral pH, high moisture content, and the abundance of readily degradable nutrients [65]. Once harvested, these factors promote biochemical changes and rapid microbial growth, which promote the deterioration of both nutritional and sensory qualities. Specifically, the breakdown of nitrogen-containing compounds results in the accumulation of volatile substances such as trimethylamine and ammonia, leading to off-flavors and unpleasant odors that diminish product quality and consumer acceptance [62].

Moreover, blue foods being rich in PUFAs are particularly susceptible to lipid oxidation, which can lead to the development of undesirable odors, flavor deterioration, nutrient degradation, the formation of potentially harmful compounds, and color changes. The initial decline in fish freshness is primarily due to the activity of endogenous enzymes and chemical reactions, whereas microbial metabolism plays a more prominent role in the later stages of spoilage. Several factors, including the distance between harvesting sites and processing facilities, storage temperature, and processing techniques, critically influence the quality and rate of deterioration of blue foods. Both intrinsic and extrinsic factors, as well as post-harvest handling and processing practices, can significantly influence the physicochemical and microbiological characteristics of blue food products, either positively or negatively [66]. To minimize spoilage during storage, processing, and distribution, strict compliance with good hygienic practices (GHP), hazard analysis and critical control points (HACCP), and good manufacturing practices (GMP) is crucial. Numerous preservation strategies have been employed to prolong the freshness and shelf life of blue foods. Recently, non-thermal approaches, particularly CP technology, have gained prominence due to the rising consumer demand for minimally processed blue food products. Notably, CP is most effective when applied to blue foods at peak freshness to help retain their desirable quality traits.

Furthermore, plasma, commonly described as the 4th state of matter, can be formed using various energy inputs such as UV and optical radiation, electrical discharges, gamma rays, X-rays, and thermal energy all of which have the capacity to ionize gases [67]. When energy is introduced into a neutral gas, it stimulates the gas molecules, leading to the formation of charged objects like electrons and photons. Once a threshold energy level is achieved, these particles interact with gas molecules and atoms through processes such as photoionization and electron-impact ionization, ultimately forming free electrons and ions. These charged components then involve in chemical reactions that produce reactive oxygen and nitrogen species (RONS), which are mostly responsible for CPs antimicrobial capabilities. The mechanisms through which CP exerts its antimicrobial effects have been extensively detailed by Olatunde and Benjakul (2018) [62], who also noted that CP acts differently against Gram-positive and Gram-negative bacterial strains. In Gram-negative bacteria, the antimicrobial effects are mainly associated with the oxidative degradation of cellular components such as proteins and lipids, the leakage of intracellular constituents (e.g., proteins and DNA), and the irreversible compromise of the cell membrane. For Gram-positive bacteria, the effects are similar, involving oxidative damage and intracellular leakage. However, the thicker peptidoglycan layer in their cell walls influences their relative resistance to CP treatment. Applications for CP on blue foods are summarized in Table 3.

Even though the effectiveness of CP demonstrates itself in extending shelf life and reducing microbial load in blue foods, its application presents several important challenges. The high reactivity of plasma-generated species can accelerate lipid oxidation in PUFA-rich marine products, potentially leading to quality deterioration such as off-flavors, discoloration, and nutrient loss. Moreover, the variability in treatment conditions, including gas composition, voltage, and exposure time, results in inconsistent outcomes across studies, highlighting the need for standardized and optimized protocols. The interaction between CP treatment and different blue food matrices also remains insufficiently understood, particularly regarding long-term effects on texture and sensory attributes during storage. Additionally, balancing microbial inactivation with minimal quality degradation remains a critical trade-off. Therefore, future research should focus on refining process parameters, integrating CP with antioxidant strategies, and developing application-specific guidelines to maximize its effectiveness while preserving the intrinsic quality of blue food products.

### 4.2. High Pressure Processing (HPP) Technology

HPP is a non-thermal preservation technique that has been widely studied for its application to blue food products, with its adoption in the food industry continuing to grow. This innovative method effectively inactivates both enzymes and spoilage microorganisms, while maintaining essential quality parameters such as color, taste, and nutritional content. Numerous studies have highlighted HPP’s ability to significantly prolong the shelf life of blue foods, thus improving their sensory properties and microbiological safety [83]. In particular, HPP has been shown to effectively eliminate pathogenic microorganisms in products like oysters, thus lowering the risk of foodborne diseases.

Moreover, HPP induces structural alterations in proteins, typically resulting in partial unfolding. This process enables the formation of both non-covalent (hydrogen, hydrophobic, and ionic) and covalent (inter- and intramolecular) interactions during the application and release of pressure [84]. Such partial denaturation can lead to significant changes in protein functional properties, including gelation, emulsification, water-holding capacity, and foaming abilities. These functional modifications may contribute to notable improvements in the texture of certain food products. Despite extensive research on the application of HPP in various food systems, there is a lack of studies specifically investigating its effects on the proteins of blue crab meat. HPP holds potential for enhancing meat yield from crabs, as it has been effectively used for shell shucking and meat extraction in bivalves such as bay and oyster’s scallops [85]. For instance, pressure treatments ranging from 200 MPa for 3 min to 350 MPa for instantaneous exposure have resulted in up to an 18% increase in tissue yield compared to manual extraction. Similarly, Cruz-Romero et al. (2004) reported a 15.5% improvement in yield from oysters treated at 260 MPa for 3 min at 20 °C compared to untreated controls [86]. The influence of HPP on protein macromolecules is highly dependent on specific processing parameters, including temperature, pressure intensity, and duration of treatment. Furthermore, the source of the protein, whether derived from rabbit, beef, pork, or fish, also plays a crucial role in determining the outcome of HPP treatment.

Furthermore, HPP induces a reduction in product volume, a phenomenon extensively reported by [87]. This volumetric contraction occurs uniformly throughout the material, consistent with the isostatic principle, which suggests that pressure is transmitted equally regardless of the sample’s size or shape. The primary cause of this volume decrease is the disruption of molecular interactions, particularly the weaker forces such as van der Waals forces, hydrogen bonding, electrostatic interactions, and hydrophobic effects. As a result, macromolecules including polysaccharides, proteins (such as enzymes), and nucleic acids may experience conformational and functional modifications. Conversely, smaller molecules like vitamins, amino acids, and flavor compounds generally exhibit stability under HPP conditions [88]. A key advantage of HPP compared to traditional thermal preservation techniques is its capability to inactivate microorganisms while preserving the nutritional quality and sensory characteristics of foods. Proposed mechanisms for microbial inactivation by high pressure include alterations in cell morphology, disruption of membrane integrity, and protein denaturation, which collectively impair vital cellular functions and disturb homeostasis [89].

HPP has demonstrated significant effectiveness in reducing microbial populations in a variety of blue food species. Standard protocols often recommend applying 300 MPa for several minutes at ambient temperature to effectively inhibit vegetative bacterial cells in numerous food matrices [90]. This method has shown potential results in a range of shellfish and fish products. Furthermore, Truong et al. (2015) collected and evaluated several HPP studies, concluding that the technology generally contributes to microbial reduction and shelf-life enhancement, although making direct comparisons across studies is often complex [91]. For instance, Cheret et al. (2005) and Teixeira et al. (2014) both applied a pressure treatment of 400 MPa for 5 min to sea bass (*Dicentrarchus labrax*), yet observed markedly different reductions in bacterial counts 0.44 log CFU/g and 3.2 log CFU/g, respectively [92,93]. These differences were attributed to variables such as initial microbial loads and differences in pressurization rates. In addition to processing parameters, the microbial community’s composition significantly influences the success of HPP. Typically, Gram-negative bacteria exhibit better susceptibility to pressure treatments than Gram-positive bacteria.

Even though its effectiveness as a non-thermal preservation method, HPP presents several challenges that limit its broader application in blue food systems. One key limitation is the variability in treatment outcomes across different species and processing conditions, which complicates the establishment of standardized protocols. While HPP can improve texture and microbial safety, excessive pressure or improper parameter selection may lead to undesirable changes such as protein over-denaturation, texture hardening, or drip loss. Additionally, the high capital investment and operational costs associated with HPP equipment may restrict its adoption, particularly for small- and medium-scale producers. Differences in microbial resistance and initial contamination levels further contribute to inconsistent efficacy across studies. Moreover, the long-term effects of HPP on sensory attributes and nutritional quality during storage remain insufficiently explored. Therefore, future research should focus on optimizing species-specific processing conditions, improving cost-efficiency, and developing integrated preservation strategies to maximize the benefits of HPP in blue food applications.

### 4.3. Pulsed-Electric Field (PEF) Technology

PEF technology is gaining recognition as a promising non-thermal approach in the food industry, valued for its cost-efficiency and environmental sustainability. While PEF has been widely applied to liquid and semi-liquid foods, further research is necessary to enable its broader commercial adoption in the processing of blue foods. The core principle of PEF involves delivering short bursts of high-voltage electric fields ranging from nanoseconds to milliseconds between two electrodes, with field intensities spanning 0.1 to 80 kV/cm [94]. The overall processing duration depends on the number of pulses multiplied by the effective pulse length. Interest in PEF for blue food applications has increased due to its efficiency in microbial inactivation and its capacity to modify structural characteristics that improve processes such as salting. Importantly, PEF treatment has demonstrated minimal impact on sensory attributes, as evidenced in studies on Asian seabass and freshwater mussels [95]. Additionally, Cropotova et al. (2021) reported that PEF pretreatment shortened brining time and enhanced salt penetration in sea bass [96]. However, it also led to raised levels of lipid oxidation products relative to untreated controls. Beyond preservation, PEF has shown potential in enabling the extraction of nutritional and bioactive compounds. Recent studies have explored PEF applications in various species including fishbone, Pacific white shrimp (*Litopenaeus vannamei*), and raw materials from sea bream and sea bass, highlighting considerable opportunities for further development in this field [97].

Regardless of its promising applications, the use of pulsed electric field technology in blue food systems is still limited by several technical and practical challenges. One key limitation is its reduced effectiveness in solid or heterogeneous food matrices compared to liquid systems, which restricts its broader applicability to whole fish or complex blue food products. Additionally, while PEF can enhance mass transfer and microbial inactivation, it may also promote lipid oxidation in PUFA-rich blue foods, potentially affecting product quality and shelf life. The variability in processing parameters such as field strength, pulse duration, and treatment time further complicates the optimization and standardization of PEF applications across different species. Moreover, industrial-scale implementation requires significant investment in specialized equipment and integration into existing processing lines. Therefore, future research should focus on improving process control, minimizing oxidative effects, and developing hybrid preservation strategies to enhance the efficiency and applicability of PEF in blue food processing.

### 4.4. Pulsed Light and Ultraviolet Technologies

The use of UV light for food preservation was initially recognized in the 1930s and has since been extensively adopted as a disinfection method across various industries. To overcome the constraints related to continuous UV exposure, flash lamps were developed as an alternative approach for delivering UV radiation. The initial use of flash lamps dates back to the late 1970s, while the adoption of pulsed light (PL) technology for microbial inactivation emerged prominently in the 1990s [98]. Traditionally, both PL and UV treatments have been employed primarily for processing facilities, sanitizing equipment, and packaging materials. However, their application directly on food products is a more recent development that is experiencing rapid growth.

Though, UV light a part of the electromagnetic spectrum, spans wavelengths from 100 to 400 nm and is typically divided into three categories based on wavelength: UV-B (280–320 nm), UV-A (320–400 nm), and UV-C (200–280 nm), with UV-C recognized for its superior germicidal effectiveness [99]. During UV exposure, energy absorption leads to the generation of DNA photoproducts, primarily pyrimidine dimers. These covalent bonds form between adjacent pyrimidine bases on the same DNA strand, interfering with transcription and translation mechanisms, thereby impairing cellular functions and ultimately resulting in cell death [100].

**Figure 4:**
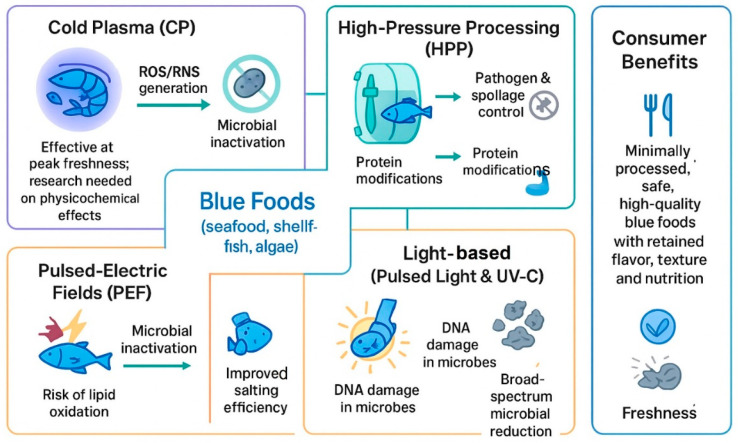
Non-thermal processing technologies for blue foods that improve microbial safety and quality while preserving freshness, flavor, texture, and nutrition.

Moreover, PL is also known by various terms such as high-intensity pulsed UV light, high-intensity broad-spectrum pulsed light, intense light pulses, pulsed UV light, intense pulsed light, or pulsed white light, employs short bursts of high-intensity, broad-spectrum light to inactivate microorganisms. This treatment covers a wavelength range from 200 to 1100 nm, which includes UV, visible, and parts of the infrared spectrum. The antimicrobial efficacy of PL is largely due to the germicidal action of the UV-C portion of the spectrum, as previously described [101]. Figure 4 shows emerging non-thermal processing technologies used in blue foods, including CP, HPP, PEFs, and light-based methods. These approaches enhance microbial safety and product quality while maintaining freshness, flavor, texture, and nutritional value.

Although their effectiveness in microbial inactivation, pulsed light and UV technologies face several limitations when applied to blue food systems. One major constraint is their limited penetration depth, which reduces efficacy in thicker or opaque food matrices such as fish fillets and shellfish. Additionally, exposure to UV radiation may induce photooxidation of lipids and proteins, particularly in PUFA-rich marine products, potentially leading to quality deterioration, including off-flavors and discoloration. The uneven surface geometry of many blue foods can also result in non-uniform treatment, creating potential zones of microbial survival. Furthermore, optimization of treatment parameters remains challenging due to variations in species, product form, and processing conditions. Industrial-scale implementation may also be limited by equipment costs and safety considerations related to UV exposure. Therefore, future research should focus on improving treatment uniformity, minimizing oxidative damage, and integrating UV and pulsed light technologies with complementary preservation methods to enhance their overall effectiveness in blue food applications.

### 4.5. Innovative Cooking and Post-Processing Approaches in Blue Food Systems

In addition to production and structuring technologies, innovative cooking approaches such as sous-vide processing have gained increasing attention in blue food systems. Sous-vide involves vacuum-sealed, low-temperature cooking, which enables precise thermal control and improved retention of moisture, flavor, and nutritional components in blue food products [102,103]. Studies have demonstrated that this technique enhances sensory attributes, including tenderness and texture, while minimizing protein denaturation and lipid oxidation [104]. Furthermore, sous-vide processing can improve the accumulation of flavor-related compounds, such as umami amino acids and nucleotides, thereby enhancing overall product quality [105]. Despite these advantages, challenges remain related to microbial safety, packaging requirements, and process optimization, particularly under anaerobic conditions [106]. Therefore, sous-vide represents a promising complementary technology for improving the quality and acceptability of both conventional and blue food analogue products.

Despite its advantages, the industrial application of sous-vide processing in blue food systems faces several important challenges. One major concern is microbial safety, particularly the risk of anaerobic pathogens such as Clostridium botulinum, which necessitates strict temperature control, validated processing conditions, and integration with additional preservation strategies such as chilling or hurdle technologies. Moreover, the requirement for specialized vacuum packaging materials and precise thermal equipment can increase operational complexity and cost, limiting scalability for large-scale production. Variability in fish species composition, fat content, and muscle structure can also influence process optimization and product consistency. In addition, while sous-vide improves sensory attributes, it does not inherently address structural limitations in alternative protein-based blue foods, which may still require advanced structuring technologies to replicate native blue food textures. Therefore, future research should focus on integrating sous-vide with other processing and preservation techniques, optimizing safety protocols, and evaluating its applicability in combination with plant-based and cultivated blue food systems to enhance both quality and scalability.

## 5. Omics-Based Insights into Blue Food Quality and Functionality

Omics-based approaches including metabolomics, proteomics, genomics, peptidomics, lipidomics, and transcriptomics have become valuable tools for assessing and verifying food products. The concept of “Foodomics,” introduced in 2009 by Cifuentes, represents a scientific discipline that integrates these technologies to study food and nutrition comprehensively [107]. Although relatively new, these advanced omics techniques have evolved swiftly and are now widely applied, either independently or synergistically, to investigate aspects such as food safety, quality, traceability, and nutritional effects on human health.

### 5.1. Proteomics

The term “proteomics,” first introduced by Marc Wilkins in 1994, refers to the comprehensive analysis of the complete set of expressed proteins within a biological system, encompassing their functions, structures, and regulatory mechanisms [108]. In the context of blue foods, proteomics serves a range of important applications, including quality assessment, authenticity verification, safety assurance, and monitoring of processing parameters. For instance, Babaheydari et al. (2016) employed proteomic methodologies, including matrix-assisted laser desorption/ionization time-of-flight/time-of-flight mass spectrometry (MALDI-TOF/TOF-MS), two-dimensional gel electrophoresis (2-DE), and database analysis, to investigate diploid and heat-stressed triploid rainbow trout larvae [109]. Their study revealed nine proteins with differential expression linked to skeletal deformities, thereby providing molecular-level insights into the etiology of these abnormalities.

Additionally, high-throughput proteomic techniques based on liquid chromatography–tandem mass spectrometry (LC-MS/MS) have been used to explore the role of phosphoproteins in grass carp muscle texture [110]. The analysis revealed 27 upregulated and 22 downregulated phosphopeptides in firmer muscles, categorized into four functional groups, contributing to a deeper understanding of protein phosphorylation’s influence on muscle firmness and overall product quality. Moreover, in another study, white-leg shrimp (*Litopenaeus vannamei*), valued for its sensory and nutritional qualities, was examined using label-free proteomics to investigate the effects of freezing on muscle proteins. Moreover, Zhang et al. (2020) utilized high-performance -MS/MS in conjunction with the UniProt Decapoda protein database to investigate the effects of sodium trimetaphosphate presoaking on shrimp during cold storage [111]. Their findings indicated that the treatment mitigated protein degradation by modulating metabolic pathways related to actins, phosphoglycerate mutase, and ribosomal proteins, thereby shedding light on the cryoprotective mechanisms involved in freezing preservation.

Moreover, proteomic methodologies have been extensively employed in the study of blue foods to authenticate species, evaluate quality, monitor processing conditions, and ensure safety. Various cutting-edge analytical platforms have been implemented in these studies, including mass spectrometry (MS), two-dimensional gel electrophoresis (2-DE), MALDI-TOF/TOF-MS, direct analysis in real-time high-resolution mass spectrometry (DART-HRMS), LC-MS/MS, and ultra-performance liquid chromatography coupled with quadrupole time-of-flight MS/MS (UPLC-QTOF-MS/MS) using SWATH acquisition. Advances in MS technologies have significantly enhanced the accuracy, sensitivity, and quantitative performance of proteomic investigations.

Although the significant advancements in proteomic technologies, several challenges limit their full application in blue food research. One major constraint is the complexity of protein extraction and identification in aquatic species, particularly due to the diversity of proteins and the lack of comprehensive, species-specific protein databases. This can lead to incomplete or biased protein characterization, especially in non-model organisms. Additionally, variations in sample preparation, analytical platforms, and data processing methods can result in inconsistencies across studies, making comparative analysis difficult. While proteomics provides valuable insights into quality, authenticity, and processing effects, translating these findings into practical industrial applications remains challenging. Furthermore, high operational costs and the requirement for specialized expertise may restrict widespread adoption. Therefore, future research should focus on improving database coverage, standardizing analytical workflows, and integrating proteomics with other omics approaches to enhance its applicability in blue food systems.

### 5.2. Metabolomics

First introduced in 1998, the field of metabolomics has since played a crucial role in clarifying the complete profile of metabolites within cells, tissues, or organisms under various conditions. When combined with proteomic and genomic data through genome-scale metabolic network modeling, metabolomics supports targeted research applications [112]. In the area of blue food products, the probability of adulteration rises during processing steps such as filleting, which often removes key morphological identifiers. To address this challenge, metabolomics has proven to be an effective analytical approach, enabling species differentiation, distinguishing between wild and farmed varieties, and identifying regional variations within the same species.

Furthermore, compared to proteomic approaches, metabolomics typically requires fewer samples, allows for faster sample processing, and involves fewer complex data interpretation due to the reduced number of metabolite classes. This enables broader applicability without the need to adjust experimental design for different animal models [113]. A simple and efficient method for fish species identification involves a one-step pretreatment using 0.1 M trifluoroacetic acid, followed by analysis through matrix-assisted laser desorption/ionization mass spectrometry (MALDI-MS). This technique enables reliable differentiation among ten fish species, offering high reproducibility and time efficiency [114]. Another rapid authentication strategy employs a Mini 11 mass spectrometer combined with ambient ionization and desorption electrospray ionization mass spectrometry (DESI-MS) to distinguish five fish species using small sample sizes (approximately 1 g). This method helps as a viable alternative to infrared spectroscopy for on-site species real-time verification. Furthermore, Chatterjee et al. (2019) applied high-resolution mass spectrometry (HRMS)-based metabolomics alongside chemometric modeling to authenticate shrimp species and determine their geographic origins, successfully identifying 34 unique biomarkers [115]. However, to improve the robustness and accuracy of such models, it is essential to broaden the reference database by including a wider range of species and sampling locations. Additionally, MALDI-TOF-MS paired with Mass-UP software has been employed to identify biomarkers capable of distinguishing Sparus aurata sourced from two aquaculture facilities on Madeira Island. Specifically, 17 unique peaks were associated with samples from Caniçal, while 18 were linked to Ribeira Brava, enabling accurate origin determination [116].

Likewise, several techniques are utilized for the authentication of blue foods, with metabolomics being one of the most widely adopted methods for species identification and determining geographical origin. Beyond these functions, metabolomics has also been applied to monitor the metabolic changes associated with protein degradation in grass carp caused by spoilage bacteria [117]. In samples inoculated with Pseudomonas putida, a general downregulation of various amino acids was observed. However, specific metabolites such as (S)-3-methyl-2-oxopentanoic acid and urocanate were significantly upregulated by 1.93- and 1.75-fold, respectively. Furthermore, when *P. putida* and *Shewanella putrefaciens* were co-inoculated, the spoilage process was enhanced, resulting in notable alterations in the metabolomic profile of the fish samples.

Even with its growing utility, metabolomics in blue food research faces several important limitations. One major challenge is the high sensitivity of metabolite profiles to environmental, biological, and processing variations, which can complicate data interpretation and reduce reproducibility across studies. Additionally, the lack of comprehensive and standardized metabolite databases for many aquatic species limits the accuracy of compound identification and comparative analysis. While metabolomics offers powerful tools for authentication and quality assessment, translating these findings into routine industrial applications remains challenging due to the complexity of data analysis and the need for advanced instrumentation. Furthermore, variability in analytical platforms and chemometric approaches can lead to inconsistent results. Therefore, future research should focus on expanding reference databases, standardizing analytical protocols, and integrating metabolomics with other omics approaches to improve reliability and practical applicability in blue food systems.

### 5.3. Lipidomics

Lipidomics, which influences high-performance and HRMS, has been applied to investigate the composition and biological roles of lipids in complex matrices and across a range of organisms. In recent years, its use in blue food research has expanded significantly. It has been effectively employed to distinguish between species, differentiate wild from farmed varieties, verify species identity, and assess both nutritional content and product quality [118]. For example, a study using hydrophilic interaction chromatography–mass spectrometry analyzed lipid compositions in commonly consumed salmonid species, such as Atlantic salmon (*Salmo salar*), king salmon (*Oncorhynchus tshawytscha*), and rainbow trout (*Oncorhynchus mykiss*) [119]. Specific phospholipids *m*/*z* 834.8 (PC 36:0) and *m*/*z* 802.8 (phosphatidylcholine, PC 34:2) were identified as molecular markers capable of differentiating salmon species at a high level of precision, surpassing traditional physical and genetic approaches.

Furthermore, differentiating between wild and farmed fish based solely on external morphology is often challenging due to their similar appearance. However, triacylglycerol (TAG) profiles, which reflect differences in dietary intake, have emerged as promising indicators for distinguishing between them. In this context, Maluly et al. (2019) introduced a fast and accurate method combining thermal imprinting with ambient sonic-spray ionization mass spectrometry to extract and analyze TAGs from the muscle and skin tissues of Salmonidae species [120]. This technique serves as a useful tool for verifying product authenticity and identifying origin, particularly when variations in diet are involved. Furthermore, ultra-performance liquid chromatography coupled with triple time-of-flight tandem mass spectrometry (UPLC-Triple TOF-MS/MS) has been employed to conduct an in-depth lipid analysis of four economically significant shrimp species including northern-mauxia shrimp, southern rough-shrimp, white-leg shrimp, and oriental river prawn [121]. Principal component analysis of the lipid profiles allowed for effective species differentiation and contributed to the establishment of a detailed lipid database aimed at supporting authentication and quality evaluation in blue food products.

**Figure 5:**
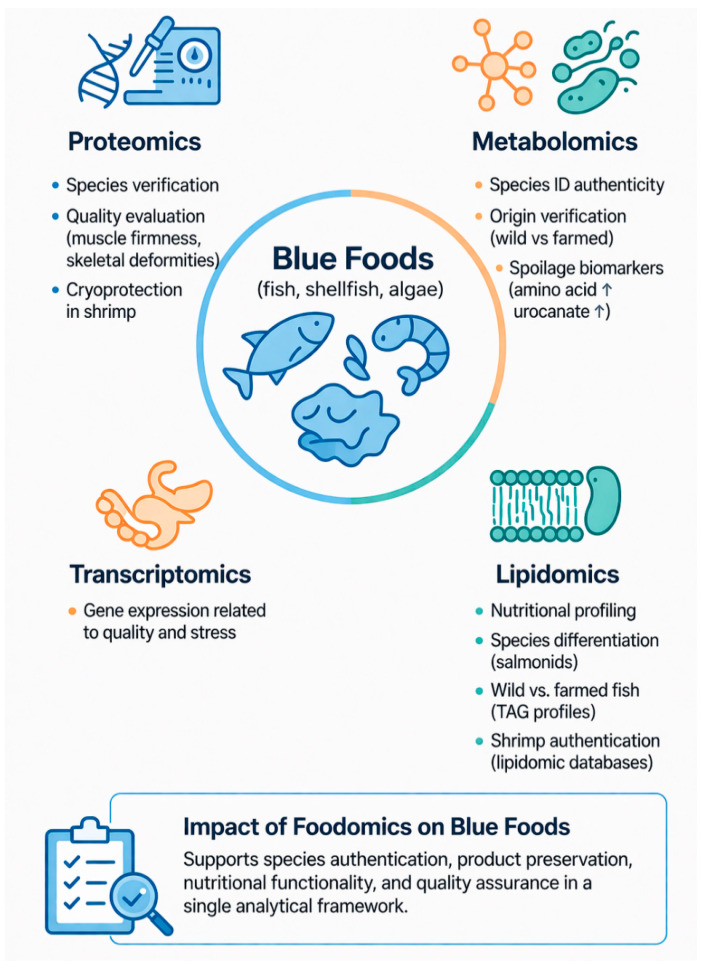
Application of foodomics approaches including proteomics, metabolomics, genomics, transcriptomics, and lipidomics in blue foods to support species authentication, traceability, quality evaluation, nutritional profiling, and product preservation within a unified analytical framework.

Moreover, the swift progress in MS-based technologies, along with developments in bioinformatics, has significantly contributed to the expanding use of lipidomics in the study of blue foods. In recent years, lipidomics has been applied to a range of objectives, such as identifying species and assessing both nutritional value and overall product quality. Its strength lies in the ability to profile the entire lipidome, offering a detailed and quantitative overview of lipid composition within a single analytical framework. Figure 5 represents the role of foodomics in blue foods through tools such as proteomics, metabolomics, genomics, transcriptomics, and lipidomics. These approaches enable species authentication, traceability, quality assessment, nutritional profiling, and product preservation.

In spite of the rapid advancements in lipidomics, several challenges limit its full potential in blue food research and industry applications. One key limitation is the high complexity and variability of lipid profiles, which are strongly influenced by factors such as diet, environment, and processing conditions, making standardization and comparison across studies difficult. Additionally, the identification and quantification of lipid species often depend on the availability of comprehensive lipid databases, which remain incomplete for many aquatic organisms. While lipidomics provides valuable insights into nutritional quality and authenticity, translating these findings into routine quality control or regulatory frameworks remains challenging due to the need for advanced instrumentation and specialized data analysis. Furthermore, discrepancies in analytical methodologies and data interpretation can affect reproducibility. Therefore, future research should focus on expanding lipid databases, harmonizing analytical workflows, and integrating lipidomics with other omics approaches to enhance its reliability and applicability in blue food systems.

## 6. Regulatory Considerations for Cultivated Blue Foods

Cell-cultivated blue food production must comply with regulatory requirements established under the Food Safety Modernization Act (FSMA), which emphasizes risk-based preventive control systems. According to the U.S. Food and Drug Administration (FDA), both international and domestic and food facilities registered under Section 415 of the Federal Drug, Food, and Cosmetic Act are required to adhere to FSMA regulations, including the implementation of preventive controls and updated Current Good Manufacturing Practices (CGMPs), unless exemptions apply. Traditionally, the FDA has overseen most blue food products, whereas catfish (*Siluridae*), along with other meat products, fall under the regulatory authority of the United States Department of Agriculture (USDA) [122].

Moreover, cell-cultivated blue food production is categorized as a novel or alternative food system, making labeling a critical component of regulatory compliance. Establishing clear and standardized terminology is essential to ensure transparency and support consumer understanding. A comprehensive study on blue food labeling demonstrated that the terms “cell-cultivated blue food” and “cell-cultured blue food” satisfy regulatory naming criteria [123]. When these labels were applied to products such as frozen Atlantic salmon, a majority of participants were able to distinguish them from conventionally labeled products, with 60.1% identifying “cell-cultivated” and 58.9% recognizing “cell-cultured” as distinct from “farm-raised” and “wild-caught” alternatives [123].

Moreover, the development of reliable analytical tools, including rapid detection systems and validated testing kits, is essential to ensure the safety of cell-cultivated blue food products. In particular, robust methods are required to evaluate allergenicity in blue foods, including those produced through cellular aquaculture. Such assessments must consider not only the cultivated cells but also additional components involved in production, such as biomaterial scaffolds [124,125]. Computational (in silico) approaches can be employed to analyze sequence homology and identify structural similarities between newly expressed proteins and known allergens [126]. Furthermore, established experimental methods approved by regulatory authorities such as the European Food Safety Authority (EFSA) and the U.S. FDA include pepsin digestion assays and immunochemical cross-reactivity testing using Immunoglobulin E (IgE) from the serum of allergic individuals [127,128].

Traceability will also play a critical role in the regulation of cell-cultivated blue food products, similar to its importance in conventional meat systems. The traditional blue food supply chain is often highly fragmented, with limited transparency between harvesting and final consumption. In contrast, cell-cultivated blue foods offer greater traceability, as production occurs under controlled conditions, allowing products to be tracked more effectively back to their origin.

## 7. Conclusions and Future Perspectives

This review article demonstrates that although multiple emerging technologies are advancing blue food systems, their performance across key dimensions such as scalability, cost-efficiency, structural fidelity, sensory realism, and nutritional equivalence remains highly heterogeneous. Cellular (cultivated) blue food systems offer the closest biological and nutritional match to conventional blue food but are limited by low technology readiness, high production costs, and scale-up challenges. In contrast, plant-based systems are technologically mature and commercially scalable, yet they struggle to replicate the complex flake-like structure, marine flavor, and omega-3 composition of blue foods. Fermentation-based approaches provide strong potential for targeted nutrient and functional ingredient production but remain constrained by economic and sensory limitations. Similarly, structuring technologies exhibit complementary strengths, with extrusion enabling industrial-scale production, while electrospinning, wet spinning, and 3D printing offer higher structural precision but limited scalability. Non-thermal preservation technologies improve safety and quality retention but require optimization to balance microbial inactivation with oxidative stability. In parallel, omics approaches provide powerful tools for authentication and quality assessment, although their industrial implementation remains limited by cost, standardization, and data complexity. Collectively, these findings indicate that no single technology currently fulfills all requirements for next-generation blue food systems.

**Figure 6:**
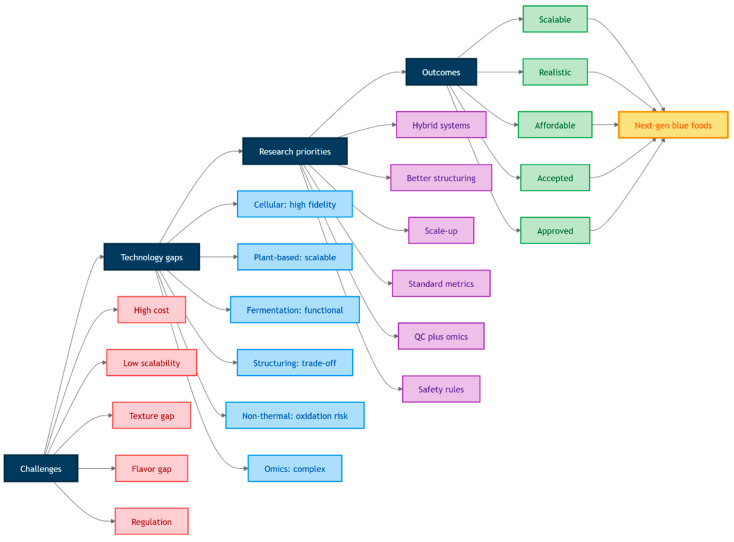
Simplified roadmap for emerging blue food technologies. The diagram outlines the transition from key challenges and technology gaps to research priorities and future outcomes, emphasizing integrated strategies to achieve scalable and sustainable next-generation blue food systems.

Future progress in blue food innovation will depend on integrating complementary technologies rather than relying on isolated approaches. Hybrid systems combining plant-based, fermentation, and cellular platforms, alongside advanced structuring techniques, are likely to play a central role in achieving both scalability and product realism. Greater emphasis is needed on standardized evaluation frameworks, including technology readiness level (TRL), CAPEX/OPEX, sensory performance, and nutritional equivalence, to enable meaningful comparison and guide industrial adoption. In addition, optimizing non-thermal and post-processing technologies, including innovative cooking methods such as sous-vide, will be critical for improving product quality and consumer acceptance. Advances in omics technologies should focus on improving database coverage, reducing analytical complexity, and enabling integration into routine quality control systems. Furthermore, the development of clear regulatory pathways, safety validation strategies, and transparent labeling frameworks will be essential to support commercialization and build consumer trust. Ultimately, interdisciplinary collaboration between academia, industry, and policymakers will be key to translating these emerging technologies into scalable, sustainable, and market-ready blue food solutions. Figure 6 illustrates a conceptual roadmap for emerging blue food technologies, outlining the progression from current challenges and technological limitations to research priorities and future outcomes. The framework highlights key barriers such as cost, scalability, sensory limitations, oxidative instability, and regulatory uncertainty, and emphasizes the role of integrated strategies including hybrid production systems, advanced structuring approaches, process optimization, and omics-based quality control in enabling scalable, cost-effective, and market-ready next-generation blue food systems.

## Figures and Tables

**Table 2:** Summary of key structuring technologies used in the development of blue food analogues.

Structuring Technology	Description/Principle	Technology Readiness Level (TRL) *	Scalability	CAPEX/OPEX	Structural Fidelity (Fish Texture)	Applications in Blue Food Analogues	Advantages	Challenges/Limitations	References
Extrusion	High shear, temperature, and pressure processing of protein hydrocolloid mixtures through a screw barrel and die system; formation of fibrous matrices via protein unfolding, aggregation, and flow-induced alignment under high- or low-moisture regimes	High	High (industrial scale)	Medium	Moderate-High (fibrous but limited flake structure)	• Fish analogues: High-moisture extrusion widely used to produce fibrous plant-based fillet-like textures • Industrial production: Predominant industrial-scale texturization method for fish analogues • Protein sources: Pulse proteins, cereal proteins, and microalgae proteins extruded to generate fish-like textures	• Scalability: Established industrial equipment enables high throughput and continuous production • Texture production: Effective at producing macroscopic anisotropic fibrous textures resembling muscle through combined heat and shear • Commercial viability: Well-established technology with proven industrial applications	• Microstructure precision: Struggles to reproduce delicate flake/lamellar fish microstructures without additional processing • Ingredient sensitivity: Heat/shear can degrade heat-labile nutrients or pigments from algal/blue sources • Sensory limitations: Matching bite, juiciness, and flavor of blue food often requires hybrid approaches or post-processing	[50,51,52]
Electrospinning	Bottom-up method using high electric field to stretch polymer/protein solutions into ultrafine nano- and microfibers; solution is ejected from a nozzle and collected as nonwoven mats or oriented bundles depending on collector design	Low–Medium	Low (lab to pilot scale)	High	High (nano-scale muscle mimicry)	Scaffold mimicry: Edible scaffolds replicating fibrillar fish muscle networks. Cultivated blue food: Substrates enabling cell attachment in cultivated blue food. Microcarriers: Edible carriers supporting scalable cell culture. Texture enhancement: Structural elements improving mouthfeel.	Precision control: Enables fine regulation of fiber diameter and orientation at micro/nano scale. Biocompatibility: Produces food-grade scaffolds suitable for cell culture. Structural mimicry: Replicates extracellular matrix-like structures of fish muscle. Versatility: Applicable to diverse protein and biopolymer solutions.	Scale-up challenges: Industrial-scale production remains difficult. Equipment complexity: Needs specialized high-voltage systems and controlled settings. Solvent considerations: Often relies on organic solvents that must be fully removed for food use. Cost: More expensive than conventional processing.	[53,54,55]
3D Printing/Bioprinting	Additive manufacturing technology that deposits material layer-by-layer to create three-dimensional structures; uses food-grade inks/pastes containing proteins, hydrocolloids, and other ingredients to build complex geometries with precise spatial control.	Medium	Low-Medium	High	High (complex geometry but limited fine fibers)	Complex geometries: Produces intricate fish-like structures Algal protein integration: Uses algal proteins for sustainable blue food analogues Customized products: Enables personalized nutrition and portion control Hybrid structures: Combines with other technologies for improved texture and functionality	Design flexibility: Allows complex geometries and customization Precision placement: Accurate positioning of ingredients and functional components Sustainable materials: Incorporates novel proteins like microalgae Waste reduction: On-demand production minimizes food waste Personalization: Enables tailored nutrition and sensory properties	Material limitations: Printable food materials limited by rheology Speed constraints: Slower production compared to conventional methods Resolution limits: Challenging to achieve fine structural details Post-processing needs: Additional treatments required for optimal texture and stability Equipment costs: High initial investment in specialized 3D printers	[56,57,58,59]
Wet Spinning	Continuous fiber formation where polymer/protein solutions (5–20% *w*/*w*) are extruded through spinnerets into a coagulation bath; Fibers form via chemical or physical coagulation, with diameters of 10–100 μm; Process includes solution preparation, spinning, coagulation, washing, and collection.	Medium	Medium-High	Medium	High (aligned fiber bundles)	Continuous fiber scaffolds: Fibers for fish muscle mimicry and structural applications Muscle fiber simulation: Aligned bundles resembling natural muscle architecture Hybrid applications: Integration with other structuring methods for multi-scale constructs Marine biopolymer processing: Using blue food waste (chitin, collagen) for structured materials Scaffold production: Edible scaffolds for cultivated blue food applications	Continuous production: Long fibers suitable for industrial-scale processing Fiber alignment: Control over orientation and bundle formation via spinneret design Scalability potential: Adaptable to large-scale processing with multiple spinnerets Material versatility: Compatible with diverse protein and biopolymer solutions Waste valorization: Utilizes marine waste as feedstock for circular economy Mechanical properties: Fibers with good tensile strength and elasticity	Process complexity: Requires strict control of coagulation chemistry, temperature, and pH Chemical considerations: Coagulation baths may introduce residues needing removal for food safety Limited structural diversity: Mainly produces fibrous structures with less complexity than 3D printing Downstream processing: Additional steps needed for washing, assembly, and product integration Environmental concerns: Waste streams from coagulation baths raise sustainability issues Equipment requirements: Specialized spinning and coagulation systems are necessary	[54,55,60,61]

* Abbreviations: TRL, Technology Readiness Level; CAPEX, Capital Expenditure; OPEX, Operational Expenditure.

**Table 3:** Use of CP alone and in combination with antioxidants in blue foods and their products.

References	Type of CP	Plasma Parameters	Working Gas	Blue Food/Blue Food Products	Findings
[68]	IB-DBD	60 s, 50 Hz and 60 V	Ambient air	Slices of *Scomber japanicus* (chub mackerel	Plasma treatment extends sliced product shelf life from 6 to 14 days by reducing protein degradation and improving tissue structure.
[69]	IB-DBD	2.5–10 min, 80 kVRMS	Oxygen/argon mixture (10:90)	Slices of *Lates calcarifer* (Asian-sea bass)	Slices treated with CP for ≥5 min showed an extended shelf life of up to 12 days at 4 °C, though prolonged exposure increased lipid oxidation.
[70]	IB-DBD	5 min, 80 kVRMS	Gas A: argon and oxygen (10:90); Gas B: carbon dioxide, argon, and oxygen (60:30:10)	Slices of *Lates calcarifer* (Asian sea bass)	CP treatment extended the shelf life of Atlantic salmon belly slices to 12 days with gas A and 15 days with gas B, versus 6 days for controls. Gas B also reduced TVB-N and TMA levels, though CP increased protein and lipid oxidation regardless of gas type.
[71]	DBD	10 min, 500 Hz, 40 kV	Ambient air	*Litopenaeus vannamei* (White shrimp)	CP treatment reduced microbial loads, extending shelf life to 14.07 days vs. 9.78 days in controls at 5 ± 1 °C. It also improved physicochemical traits and sensory attributes during storage.
[71]	IB-DBD	5 min, 80 kV or 70 kV	Ambient air	Fillets of *Scomber scombrus* (mackerel)	CP-treated samples showed significantly lower microbial loads than controls, with treatment intensity and duration affecting both microbial inactivation efficiency and lipid oxidation levels.
[72]	IB-DBD	5 min, 80 kV or 70 kV	Ambient air	Fillets of *Clupea harengus* (herring)	CP treatment reduced microbial loads across all tested groups, including mesophilic, psychrotrophic, lactic acid bacteria, Pseudomonas, and *Enterobacteriaceae*. The lowest voltage preserved quality best, with minimal oxidation and color changes.
[73]	IB-DBD	2.5–10 min, 80 kVRMS	Oxygen mixture/Argon (10:90)	Slices of *Lates calcarifer* (Asian seabass)	CP treatment significantly reduced microbial counts in slices, with longer exposures yielding greater reductions but also increasing protein and lipid oxidation.
[74]	DBD	2.5–15 min (treatment time) and 30–50 kV (voltage)	Ambient air	Fillets of *Trichiurus japonicus* (hairtail fish)	The effectiveness of CP in inhibiting endogenous enzyme activity depended on both treatment duration and applied voltage.
[75]	DBD	30–300 s for 50 kV	Ambient air	Fillets of *Trichiurus lepturus* (hairtail fish)	CP treatment induced a time-dependent decline in crude enzyme activity, coinciding with a threefold increase in protein carbonyls and a decrease in total sulfhydryl groups. Additionally, it significantly enhanced muscle protein texture, water-holding capacity, and color.
[76]	DBD	15–300 s (treatment time), 60 kV	Ambient air	Mantle of *Argentinus ilex* (squid)	Protease activity decreased progressively with longer CP exposure, with a 64% reduction after 240 s. CP treatment also significantly improved the color, texture, and water-holding capacity of squid gel compared to controls.
[77]	DBD	30 w and 0.3 A, 0–30 min (treatment time)	Argon	Natural actomyosin (NAM) from *Nemipterus bleekeri* (threadfin bream)	CP treatment progressively reduced total sulfhydryl content, Ca^2+^-ATPase activity, and NAM solubility, while increasing protein carbonyl levels. It also induced biophysical changes and NAM aggregation, potentially enhancing gelation properties in threadfin bream surimi.
[78]	DBD	A treatment of 12 kV and 18 kHz was applied for helium gas, while 22 kV and 32 kHz were used for argon. Treatment durations of 4, 6, and 10 min were employed.	Argon and helium	Fish nuggets inoculated with *Listeria innocua* and *Staphylococcus aureus* bacteria	Antimicrobial efficacy of helium- and argon-based CP treatments increased with longer exposure times against targeted bacterial strains.
[79]	JP/DBD	JP: 3.0 L/min for 30 s, 50 Hz, 20 kV; DBD: 6.5 L/min for 300 s, 75 Hz, 12.8 kV	Ambient air	Commercial fish balls inoculated with *Psychrobacter glacincola* 38-1, *Brochothrix thermosphacta* 38-2, and *Pseudomonas fragi* 38-8, treated with CP combined with antioxidants	Both plasma treatments inhibited bacterial growth, with dielectric barrier discharge (DBD) showing stronger antimicrobial effects than jet plasma (JP).
[80]	IB-DBD	5 min, 80 kV	Carbon dioxide, argon, and oxygen (60:30:10)	Slices of *Lates calcarifer* (Asian seabass)	Combining CP treatment with 400 mg/kg liposomally encapsulated ethanolic coconut husk extract (Lie-ECHE) enhanced antimicrobial efficacy, extending shelf life to 18 days versus 15 days with CP alone.
[69]	IB-DBD	5 min, 80 kV	5 min, oxygen and argon (10:90)	Slices of *Lates calcarifer* (Asian seabass fish)	Incorporating antioxidants like ECHE and ascorbic acid with CP treatment reduced CP-induced protein and lipid oxidation, with ECHE offering the strongest protection, extending shelf life to 15 days at 4 °C.
[81]	IB-DBD	10 min, 16 kVRMS	Argon/air (80:20) and Argon/air (80:20)	*Litopenaeus vannamei* (Pacific white shrimp)	Pre-treatment with 1% Chamuang leaf extract followed by CP using an 80:20 argon-to-air mix effectively preserved shrimp quality for 15 days at 4 °C.
[69]	IB-DBD	10 min, 80 kVRMS	Argon, oxygen and carbon dioxide (30:10:60)	Slices of *Lates calcarifer* (Asian seabass)	CP combined with 400 ppm liposomally encapsulated coconut husk extract effectively extended the shelf life of Asian seabass slices to over 18 days at 4 °C.
[82]	IB-DBD	5 min, 16 kVRMS	Oxygen and argon (10:90)	Slices of *Lates calcarifer* (Asian seabass fish)	Combining CP treatment with 0.1% or 0.2% chito-oligosaccharides reduced CP-induced protein and lipid oxidation, extending the shelf life of Asian seabass slices to 12 days at 4 °C.

Abbreviations: JP: Jet plasma; IB-DBD: In bag dielectric barrier discharge; CP: Cold plasma; DBD: Dielectric barrier discharge.

## Data Availability

No new data were created or analyzed in this study. Data sharing is not applicable to this article.

## Abbreviations

The following abbreviations are used in this manuscript:

2-DE	Two-Dimensional Gel Electrophoresis
CAD	Computer-Aided Design
CP	Cold Plasma
DBD	Dielectric Barrier Discharge
DESI-MS	Desorption Electrospray Ionization Mass Spectrometry
DART-HRMS	Direct Analysis in Real-Time High-Resolution Mass Spectrometry
FAO	Food and Agriculture Organization
GHP	Good Hygienic Practices
GMP	Good Manufacturing Practices
HACCP	Hazard Analysis and Critical Control Points
HRMS	High-Resolution Mass Spectrometry
IB-DBD	In-Bag Dielectric Barrier Discharge
LC-MS/MS	Liquid Chromatography–Tandem Mass Spectrometry
MALDI-MS	Matrix-Assisted Laser Desorption/Ionization Mass Spectrometry
MALDI-TOF/TOF-MS	Matrix-Assisted Laser Desorption/Ionization Time-of-Flight/Time-of-Flight Mass Spectrometry
MS	Mass Spectrometry
NAM	Natural Actomyosin
OECD	Organisation for Economic Co-operation and Development
PEF	Pulsed Electric Field
PL	Pulsed Light
PUFAs	Polyunsaturated Fatty Acids
RNS	Reactive Nitrogen Species
ROS	Reactive Oxygen Species
SCP	Single-Cell Proteins
SWATH	Sequential Window Acquisition of All Theoretical Mass Spectra
TAG	Triacylglycerol
TVB-N	Total Volatile Basic Nitrogen
UPLC-QTOF-MS/MS	Ultra-Performance Liquid Chromatography Quadrupole Time-of-Flight Tandem Mass Spectrometry
UV	Ultraviolet

## References

[1] Koehn J.Z., Allison E.H., Golden C.D., Hilborn R. (2022). The role of seafood in sustainable diets. Environ. Res. Lett..

[2] Farmery A.K., Allison E.H., Andrew N.L., Troell M., Voyer M., Campbell B., Eriksson H., Fabinyi M., Song A.M., Steenbergen D. (2021). Blind spots in visions of a “blue economy” could undermine the ocean’s contribution to eliminating hunger and malnutrition. One Earth.

[3] Henchion M., Hayes M., Mullen A.M., Fenelon M., Tiwari B. (2017). Future Protein Supply and Demand: Strategies and Factors Influencing a Sustainable Equilibrium. Foods.

[4] FAO (2020). The State of World Fisheries and Aquaculture 2020. Sustainability in Action.

[5] Pomeroy R., Parks J., Mrakovcich K.L., LaMonica C. (2016). Drivers and impacts of fisheries scarcity, competition, and conflict on maritime security. Mar. Policy.

[6] Cherry D. Thai Union Joins $60 Million Financing Round in Cell-Based Seafood Group. Intrafish.

[7] Nirmal N., Anyimadu C.F., Khanashyam A.C., Bekhit A.E.d.A., Dhar B.K. (2024). Alternative Protein Sources: Addressing Global Food Security and Environmental Sustainability. Sustain. Dev..

[8] Bry-Chevalier T., AgroParisTech-INRAE B. (2024). Comparing the potential of meat alternatives for a more sustainable food system. OSF Prepr..

[9] Cardoso N., dos Santos M., Fernandes R.R., Manasfi M., Lopes G., Zanette G.B. ‘Cell-based seafood’: Revisão da literatura e novas perspectivas. Proceedings of the I Simpósio de Bolsistas da FIPERJ.

[10] Magdum A.B., Shinde K.V., Jadhav N.K., Jagtap M.A., Patil S.A., Nimbalkar M.S. (2026). Plant-based proteins: Functional, nutritional, and technological advances for sustainable food systems. Eur. Food Res. Technol..

[11] Li Y., Xiang N., Zhu Y., Yang M., Shi C., Tang Y., Sun W., Sheng K., Liu D., Zhang X. (2024). Blue source-based food alternative proteins: Exploring aquatic plant-based and cell-based sources for sustainable nutrition. Trends Food Sci. Technol..

[12] Lisboa H.M., Pasquali M.B., dos Anjos A.I., Sarinho A.M., de Melo E.D., Andrade R., Batista L., Lima J., Diniz Y., Barros A. (2024). Innovative and sustainable food preservation techniques: Enhancing food quality, safety, and environmental sustainability. Sustainability.

[13] Costello C., Cao L., Gelcich S., Cisneros-Mata M., Free C.M., Froehlich H.E., Golden C.D., Ishimura G., Maier J., Macadam-Somer I. (2020). The future of food from the sea. Nature.

[14] Organisation for Economic Co-Operation and Development (OECD) (2020). The Food and Agriculture Organization (FAO) of the United Nations. OECD-FAO Agricultural Outlook 2020–2029.

[15] FAO (2018). The State of World Fisheries and Aquaculture. Meeting the Sustainable Development Goals.

[16] Post M.J. (2014). Cultured beef: Medical technology to produce food. J. Sci. Food Agric..

[17] World Health Organization (2015). People’s Republic of China Health System review.

[18] (2020). Singapore Becomes First Country to Approve Lab-Grown Meat. London Daily.

[19] Telesetsky A. (2023). Cellular mariculture: Challenges of delivering sustainable protein security. Mar. Policy.

[20] Vegconomist (2022). Bluu Seafood: “By 2025 cultivated seafood can be expected to appear in supermarkets”. Vegconomist—The Vegan Business Magazine.

[21] Rischer H., Szilvay G.R., Oksman-Caldentey K.-M. (2020). Cellular agriculture—Industrial biotechnology for food and materials. Curr. Opin. Biotechnol..

[22] Kadim I.T., Mahgoub O., Baqir S., Faye B., Purchas R. (2015). Cultured meat from muscle stem cells: A review of challenges and prospects. J. Integr. Agric..

[23] Barzee T.J., El Mashad H.M., Cao L., Chio A., Pan Z., Zhang R. (2022). Cell-cultivated food production and processing: A review. Food Bioeng..

[24] Colombo S.M., Roy K., Mraz J., Wan A.H., Davies S.J., Tibbetts S.M., Øverland M., Francis D.S., Rocker M.M., Gasco L. (2023). Towards achieving circularity and sustainability in feeds for farmed blue foods. Rev. Aquac..

[25] Awuchi C.G., Chukwu C.N., Iyiola A.O., Noreen S., Morya S., Adeleye A.O., Twinomuhwezi H., Leicht K., Mitaki N.B., Okpala C.O.R. (2022). Bioactive Compounds and Therapeutics from Fish: Revisiting Their Suitability in Functional Foods to Enhance Human Wellbeing. Biomed. Res. Int..

[26] Khan I., Hussain M., Jiang B., Zheng L., Pan Y., Hu J., Khan A., Ashraf A., Zou X. (2023). Omega-3 long-chain polyunsaturated fatty acids: Metabolism and health implications. Progress. Lipid Res..

[27] Peinado I., Miles W., Koutsidis G. (2016). Odour characteristics of seafood flavour formulations produced with fish by-products incorporating EPA, DHA and fish oil. Food Chem..

[28] Zou X., Khan I., Wang Y., Hussain M., Jiang B., Zheng L., Pan Y., Hu J., Khalid M.U. (2024). Preparation of medium- and long-chain triacylglycerols rich in n-3 polyunsaturated fatty acids by bio-imprinted lipase-catalyzed interesterification. Food Chem..

[29] Zou X., Hussain M., Khan I., Wang Y., Jiang B., Zheng L., Pan Y., Hu J., Ashraf A. (2024). Bio-imprinted lipase-catalyzed production of medium- and long-chain structured lipids rich in n-3 polyunsaturated fatty acids by acidolysis. Food Biosci..

[30] Hussain M., Bisht A., Khan I., Chaudhary M.N., Kanwal N., Khalid M.U., Yiasmin M.N., Hussain A., Zou X. (2025). Enhanced stability of n-3 PUFAs rich structured lipids via spray-dried microencapsulation with tailored wall materials. Sustain. Food Technol..

[31] Khan I., Hussain M., Khan A., Jiang B., Zheng L., Hossan S., AL-Ansi W., Zou X. (2024). Novel medium-and long-chain triacylglycerols rich structured lipids enriched in n-3 polysaturated fatty acids encapsulated by spray drying: Characterization and stability. Int. J. Environ. Agric. Biotechnol..

[32] Olgunoglu I. (2017). Review on Omega-3 (n-3) Fatty Acids in Fish and Seafood. J. Biol. Agric. Healthc..

[33] Chan D.L.-K., Lim P.-Y., Sanny A., Georgiadou D., Lee A.P., Tan A.H.-M. (2024). Technical, commercial, and regulatory challenges of cellular agriculture for seafood production. Trends Food Sci. Technol..

[34] Blois M. (2022). Cell-based seafood is catching on Investments and distribution deals are signs that industry is starting to mature. C&EN.

[35] Borriello A., Pierucci A. (2025). A global comprehensive review on cultured seafood. npj Sci. Food.

[36] Smith D.J., Helmy M., Lindley N.D., Selvarajoo K. (2022). The transformation of our food system using cellular agriculture: What lies ahead and who will lead it?. Trends Food Sci. Technol..

[37] Thakur A., Sharma D., Saini R., Suhag R., Thakur D. (2024). Cultivating blue food proteins: Innovating next-generation ingredients from macro and microalgae. Biocatal. Agric. Biotechnol..

[38] Marwaha N., Beveridge M.C., Phillips M.J. (2022). Fad, food, or feed: Alternative seafood and its contribution to food systems. Front. Sustain. Food Syst..

[39] Kazir M., Livney Y.D. (2021). Plant-Based Seafood Analogs. Molecules.

[40] Ran X., Lou X., Zheng H., Gu Q., Yang H. (2022). Improving the texture and rheological qualities of a plant-based fishball analogue by using konjac glucomannan to enhance crosslinks with soy protein. Innov. Food Sci. Emerg. Technol..

[41] Zhang T., Dou W., Zhang X., Zhao Y., Zhang Y., Jiang L., Sui X. (2021). The development history and recent updates on soy protein-based meat alternatives. Trends Food Sci. Technol..

[42] Sun C., Fu J., Chang Y., Li S., Fang Y. (2021). Structure Design for Improving the Characteristic Attributes of Extruded Plant-Based Meat Analogues. Food Biophys..

[43] Samard S., Gu B.Y., Ryu G.H. (2019). Effects of extrusion types, screw speed and addition of wheat gluten on physicochemical characteristics and cooking stability of meat analogues. J. Sci. Food Agric..

[44] Gagaoua M., Pinto V.Z., Göksen G., Alessandroni L., Lamri M., Dib A.L., Boukid F. (2022). Electrospinning as a promising process to preserve the quality and safety of meat and meat products. Coatings.

[45] Dick A., Bhandari B., Prakash S. (2019). 3D printing of meat. Meat Sci..

[46] Boukid F. (2021). Plant-based meat analogues: From niche to mainstream. Eur. Food Res. Technol..

[47] Ko H.J., Wen Y., Choi J.H., Park B.R., Kim H.W., Park H.J. (2021). Meat analog production through artificial muscle fiber insertion using coaxial nozzle-assisted three-dimensional food printing. Food Hydrocoll..

[48] Cui B., Liang H., Li J., Zhou B., Chen W., Liu J., Li B. (2022). Development and characterization of edible plant-based fibers using a wet-spinning technique. Food Hydrocoll..

[49] Singh M., Trivedi N., Enamala M.K., Kuppam C., Parikh P., Nikolova M.P., Chavali M. (2021). Plant-based meat analogue (PBMA) as a sustainable food: A concise review. Eur. Food Res. Technol..

[50] Cheng Y., Meng Y., Liu S. (2024). Diversified techniques for restructuring meat protein-derived products and analogues. Foods.

[51] Gürbüz B.N., Pastrana L.M., Pereira R.N., Cerqueira M.A. (2025). Alternative protein-based meat and fish analogs by conventional and novel processing technologies: A systematic review and bibliometric analysis. Foods.

[52] Zhong C., Feng Y., Xu Y. (2023). Production of fish analogues from plant proteins: Potential strategies, challenges, and outlook. Foods.

[53] Dagès B. (2024). Novel Edible Microcarriers for the Scalable Production of Cultivated Meat. Ph.D. Thesis.

[54] Jo B., Nie M., Takeuchi S. (2021). Manufacturing of animal products by the assembly of microfabricated tissues. Essays Biochem..

[55] Kumar A., Sood A., Han S.S. (2023). Technological and structural aspects of scaffold manufacturing for cultured meat: Recent advances, challenges, and opportunities. Crit. Rev. Food Sci. Nutr..

[56] Samad A., Muazzam A., Alam A.N., Hwang Y.-H., Joo S.-T. (2026). Synergistic Effects of mTG-Induced Protein Crosslinking and Methyl Cellulose Polymer in Modulating the Quality Parameters of Hybrid Meat Patties. Appl. Sci..

[57] Alasibi S., Kazir M., Israel Á., Livney Y.D. (2024). Algal protein-based 3D-printed fish-analogs as a new approach for sustainable seafood. Curr. Res. Food Sci..

[58] Bisht B., Begum J.S., Dmitriev A.A., Kurbatova A., Singh N., Nishinari K., Nanda M., Kumar S., Vlaskin M.S., Kumar V. (2024). Unlocking the potential of future version 3D food products with next generation microalgae blue protein integration: A review. Trends Food Sci. Technol..

[59] Maxy D., Khalid N.I., Teh H.F., Kamalbatcha Z., Sulaiman R., Noh T.U., Yong Seng Ping A., Mohd Ramli S.H. (2024). Extrusion-Based 3D Printing Technology in Soy Protein-Based Meat Analogs: A Review. J. Food Process Eng..

[60] Chee P.L., Sathasivam T., Tan Y.C., Wu W., Leow Y., Lim Q.R.T., Yew P.Y.M., Zhu Q., Kai D. (2024). Nanochitin for sustainable and advanced manufacturing. Nanoscale.

[61] Karaoğlan S.Y., Darici M. (2024). Environmental Impacts/Benefits/Risk of Food Analogues. Food Analogues: Emerging Methods and Challenges.

[62] Olatunde O.O., Benjakul S. (2018). Nonthermal processes for shelf-life extension of seafoods: A revisit. Compr. Rev. Food Sci. Food Saf..

[63] Fukuda S., Kawasaki Y., Izawa S. (2019). Ferrous chloride and ferrous sulfate improve the fungicidal efficacy of cold atmospheric argon plasma on melanized Aureobasidium pullulans. J. Biosci. Bioeng..

[64] Moutiq R., Misra N., Mendonça A., Keener K. (2020). In-package decontamination of chicken breast using cold plasma technology: Microbial, quality and storage studies. Meat Sci..

[65] Viji P., Venkateshwarlu G., Ravishankar C., Srinivasa Gopal T. (2017). Role of plant extracts as natural additives in fish and fish products—A Review. Fish. Technol..

[66] DeWitt C.A.M., Oliveira A.C. (2016). Modified atmosphere systems and shelf life extension of fish and fishery products. Foods.

[67] Pankaj S.K., Wan Z., Keener K.M. (2018). Effects of cold plasma on food quality: A review. Foods.

[68] Chen J., Wang S.Z., Chen J.Y., Chen D.Z., Deng S.G., Xu B. (2019). Effect of cold plasma on maintaining the quality of chub mackerel (Scomber japonicus): Biochemical and sensory attributes. J. Sci. Food Agric..

[69] Olatunde O.O., Benjakul S., Vongkamjan K. (2019). Combined effects of high voltage cold atmospheric plasma and antioxidants on the qualities and shelf-life of Asian sea bass slices. Innov. Food Sci. Emerg. Technol..

[70] Olatunde O.O., Benjakul S., Vongkamjan K. (2020). Shelf-life of refrigerated Asian sea bass slices treated with cold plasma as affected by gas composition in packaging. Int. J. Food Microbiol..

[71] da Silva Campelo M.C., Rebouças L.d.O.S., de Oliveira Vitoriano J., Junior C.A., da Silva J.B.A., de Oliveira Lima P. (2019). Use of cold atmospheric plasma to preserve the quality of white shrimp (*Litopenaeus vannamei*). J. Food Prot..

[72] Albertos I., Martin-Diana A., Cullen P.J., Tiwari B.K., Ojha K.S., Bourke P., Rico D. (2019). Shelf-life extension of herring (*Clupea harengus*) using in-package atmospheric plasma technology. Innov. Food Sci. Emerg. Technol..

[73] Olatunde O.O., Benjakul S., Vongkamjan K. (2019). High voltage cold atmospheric plasma: Antibacterial properties and its effect on quality of Asian sea bass slices. Innov. Food Sci. Emerg. Technol..

[74] Hatab S. (2018). Effect of cold atmospheric plasma (CAP) on endogenous enzyme activity and quality parameters of hairtail (*Trichiurus japonicus*). J. Food Dairy Sci..

[75] Koddy J.K., Miao W., Hatab S., Tang L., Xu H., Nyaisaba B.M., Chen M., Deng S. (2021). Understanding the role of atmospheric cold plasma (ACP) in maintaining the quality of hairtail (*Trichiurus Lepturus*). Food Chem..

[76] Nyaisaba B.M., Miao W., Hatab S., Siloam A., Chen M., Deng S. (2019). Effects of cold atmospheric plasma on squid proteases and gel properties of protein concentrate from squid (*Argentinus ilex*) mantle. Food Chem..

[77] Panpipat W., Chaijan M. (2020). Effect of atmospheric pressure cold plasma on biophysical properties and aggregation of natural actomyosin from threadfin bream (*Nemipterus bleekeri*). Food Bioprocess. Technol..

[78] Hajhoseini A., Sharifan A., Yousefi H. (2020). Effects of atmospheric cold plasma on microbial growth of Listeria innocua and Staphylococcus aureus in ready-to-eat fish products. Iran. J. Fish. Sci..

[79] Zhang Y., Wei J., Yuan Y., Chen H., Dai L., Wang X., Yue T. (2019). Bactericidal effect of cold plasma on microbiota of commercial fish balls. Innov. Food Sci. Emerg. Technol..

[80] Olatunde O.O., Benjakul S., Vongkamjan K. (2020). Microbial diversity, shelf-life and sensory properties of Asian sea bass slices with combined treatment of liposomal encapsulated ethanolic coconut husk extract and high voltage cold plasma. LWT.

[81] Shiekh K.A., Benjakul S. (2020). Effect of high voltage cold atmospheric plasma processing on the quality and shelf-life of Pacific white shrimp treated with Chamuang leaf extract. Innov. Food Sci. Emerg. Technol..

[82] Singh A., Benjakul S. (2020). The combined effect of squid pen chitooligosaccharides and high voltage cold atmospheric plasma on the shelf-life extension of Asian sea bass slices stored at 4 °C. Innov. Food Sci. Emerg. Technol..

[83] Christensen L.B., Hovda M.B., Rode T.M. (2017). Quality changes in high pressure processed cod, salmon and mackerel during storage. Food Control.

[84] Huppertz T., Fox P.F., Kelly A.L. (2004). High pressure-induced denaturation of α-lactalbumin and β-lactoglobulin in bovine milk and whey: A possible mechanism. J. Dairy Res..

[85] Yi J., Xu Q., Hu X., Dong P., Liao X., Zhang Y. (2013). Shucking of bay scallop (*Argopecten irradians*) using high hydrostatic pressure and its effect on microbiological and physical quality of adductor muscle. Innov. Food Sci. Emerg. Technol..

[86] Cruz-Romero M., Smiddy M., Hill C., Kerry J.P., Kelly A.L. (2004). Effects of high pressure treatment on physicochemical characteristics of fresh oysters (*Crassostrea gigas*). Innov. Food Sci. Emerg. Technol..

[87] Balasubramaniam V.M., Martinez-Monteagudo S., Gupta R. (2015). Principles and Application of High Pressure--Based Technologies in the Food Industry. Annu. Rev. Food Sci. Technol..

[88] Patterson M.F. (2013). Food Technologies: High Pressure Processing. Encycl. Food Saf..

[89] Ferreira M., Almeida A., Delgadillo I., Saraiva J.A., Cunha Â (2016). Susceptibility of Listeria monocytogenes to high pressure processing: A review. Food Rev. Int..

[90] Farkas D.F., Hoover D.G. (2000). High Pressure Processing. J. Food Saf..

[91] Truong B.Q., Buckow R., Stathopoulos C.E., Nguyen M.H. (2015). Advances in High-Pressure Processing of Fish Muscles. Food Eng. Rev..

[92] Cheret R., Chapleau N., Delbarre-Ladrat C., Verrez-Bagnis V., Lamballerie M.d. (2005). Effects of high pressure on texture and microstructure of sea bass (*Dicentrarchus labrax* L.) fillets. J. Food Sci..

[93] Teixeira B., Marques A., Mendes R., Gonçalves A., Fidalgo L., Oliveira M., Saraiva J.A., Nunes M.L. (2014). Effects of High-Pressure Processing on the Quality of Sea Bass (*Dicentrarchus labrax*) Fillets During Refrigerated Storage. Food Bioprocess. Technol..

[94] Barba F.J., Koubaa M., do Prado-Silva L., Orlien V., Sant’Ana A.d.S. (2017). Mild processing applied to the inactivation of the main foodborne bacterial pathogens: A review. Trends Food Sci. Technol..

[95] Zhou Y., He Q., Zhou D. (2016). Optimization Extraction of Protein from Mussel by High-Intensity Pulsed Electric Fields. J. Food Process. Preserv..

[96] Cropotova J., Tappi S., Genovese J., Rocculi P., Laghi L., Rosa M.D., Rustad T. (2021). Study of the influence of pulsed electric field pre-treatment on quality parameters of sea bass during brine salting. Innov. Food Sci. Emerg. Technol..

[97] Franco D., Munekata P.E.S., Agregán R., Bermúdez R., López-Pedrouso M., Pateiro M., Lorenzo J.M. (2020). Application of Pulsed Electric Fields for Obtaining Antioxidant Extracts from Fish Residues. Antioxidants.

[98] Mandal R., Mohammadi X., Wiktor A., Singh A., Pratap Singh A. (2020). Applications of Pulsed Light Decontamination Technology in Food Processing: An Overview. Appl. Sci..

[99] Knuschke P., John S.M., Johansen J.D., Rustemeyer T., Elsner P., Maibach H.I. (2020). UV Exposure. Kanerva’s Occupational Dermatology.

[100] Koutchma T., Forney L.J., Moraru C.I. (2009). Ultraviolet Light in Food Technology: Principles and Applications.

[101] Gómez-Estaca J., Gómez-Guillén M.C., Montero P. (2007). High pressure effects on the quality and preservation of cold-smoked dolphinfish (*Coryphaena hippurus*) fillets. Food Chem..

[102] Russo G.L., Langellotti A.L., Buonocunto G., Puleo S., Di Monaco R., Anastasio A., Vuoso V., Smaldone G., Baselice M., Capuano F. (2023). The sous vide cooking of mediterranean mussel (*Mytilus galloprovincialis*): Safety and quality assessment. Foods.

[103] Gokoglu N. (2026). A novel cooking technique for seafood: Sous vide. Adv. Food Nutr. Res..

[104] Coşansu S., Mol S., Haskaraca G. (2022). Sous-vide cooking: Effects on seafood quality and combination with other hurdles. Int. J. Gastron. Food Sci..

[105] Li C., Lin S., Chen T., Wang S., Qian X., Chen D., Wang R., Sun N. (2025). Sous-vide cooking as a promising approach for developing high-quality salt pan shrimp products. Food Chem..

[106] Bhattacharya A., Chowdhury S., Ikbal A. (2024). Sous Vide in Seafood: A Novel Approach to Modern Processing Techniques. Chron. Aquat. Sci..

[107] Cifuentes A. (2009). Food analysis and foodomics. J. Chromatogr. A.

[108] Wilkins M.R., Appel R.D., Williams K.L., Hochstrasser D.F. (2008). Proteome Research: Concepts, Technology and Application.

[109] Babaheydari S.B., Keyvanshokooh S., Dorafshan S., Johari S.A. (2016). Proteomic analysis of skeletal deformity in diploid and triploid rainbow trout (*Oncorhynchus mykiss*) larvae. Comp. Biochem. Physiol. Part D Genom. Proteom..

[110] Chen L., Liu J., Kaneko G., Xie J., Wang G., Yu D., Li Z., Ma L., Qi D., Tian J. (2020). Quantitative phosphoproteomic analysis of soft and firm grass carp muscle. Food Chem..

[111] Zhang B., Mao J.-l., Yao H., Aubourg S.P. (2020). Label-free based proteomics analysis of protein changes in frozen whiteleg shrimp (*Litopenaeus vannamei*) pre-soaked with sodium trimetaphosphate. Food Res. Int..

[112] Baker M. (2011). Metabolomics: From small molecules to big ideas. Nat. Methods.

[113] Alfaro A.C., Young T. (2018). Showcasing metabolomic applications in aquaculture: A review. Rev. Aquac..

[114] Wang S., Pang J., Liang P. (2021). Differential proteomics analysis of Penaeus vannamei muscles with quality characteristics by TMT quantitative proteomics during low-temperature storage. J. Agric. Food Chem..

[115] Chatterjee N.S., Chevallier O.P., Wielogorska E., Black C., Elliott C.T. (2019). Simultaneous authentication of species identity and geographical origin of shrimps: Untargeted metabolomics to recurrent biomarker ions. J. Chromatogr. A.

[116] Freitas J., Silva P., Perestrelo R., Vaz-Pires P., Câmara J.S. (2022). Improved approach based on MALDI-TOF MS for establishment of the fish mucus protein pattern for geographic discrimination of Sparus aurata. Food Chem..

[117] Zhuang S., Tan Y., Hong H., Li D., Zhang L., Luo Y. (2022). Exploration of the roles of spoilage bacteria in degrading grass carp proteins during chilled storage: A combined metagenomic and metabolomic approach. Food Res. Int..

[118] Tu C.-h., Qi X.-e., Shui S.-s., Lin H.-m., Benjakul S., Zhang B. (2022). Investigation of the changes in lipid profiles induced by hydroxyl radicals in whiteleg shrimp (*Litopenaeus vannamei*) muscle using LC/MS-based lipidomics analysis. Food Chem..

[119] Yu X., Li L., Wang H., Song G., Wang J., Li S., Wang Y., Shen Q. (2020). Lipidomics study of rainbow trout (*Oncorhynchus mykiss*) and salmons (*Oncorhynchus tshawytscha* and *Salmo salar*) using hydrophilic interaction chromatography and mass spectrometry. LWT.

[120] Maluly H.D.B., de Melo Porcari A., da Silva Cunha I.B., Pacheco M.T.B., Eberlin M.N., Alberici R.M. (2019). The impacts of the raising regime of Salmon species on their triacylglycerol composition revealed by easy ambient sonic-spray ionization mass spectrometry. Food Res. Int..

[121] Sun H., Song Y., Zhang H., Zhang X., Liu Y., Wang X., Cong P., Xu J., Xue C. (2020). Characterization of lipid composition in the muscle tissue of four shrimp species commonly consumed in China by UPLC−Triple TOF−MS/MS. LWT.

[122] Food and Drug Administration (2024). Current Good Manufacturing Practices (CGMPs) for Food and Dietary Supplements.

[123] Hallman W.K., Hallman W.K. (2021). A comparison of cell-based and cell-cultured as appropriate common or usual names to label products made from the cells of fish. J. Food Sci..

[124] EFSA Panel on Dietetic Products, Nutrition and Allergies (NDA) (2014). Scientific Opinion on the evaluation of allergenic foods and food ingredients for labelling purposes. EFSA J..

[125] Baumert J., Brooke-Taylor S., Che H., Chen H., Crevel R., Houben G., Jackson L., Kyriakidis S., La Vieille S., Lee N.A. (2022). Risk Assessment of Food Allergens. Part 1: Review and Validation of Codex Alimetarius Priority Allergen List Through Risk Assessment.

[126] Ladics G.S., Cressman R.F., Herouet-Guicheney C., Herman R.A., Privalle L., Song P., Ward J.M., McClain S. (2011). Bioinformatics and the allergy assessment of agricultural biotechnology products: Industry practices and recommendations. Regul. Toxicol. Pharmacol..

[127] Ansotegui I.J., Melioli G., Canonica G.W., Caraballo L., Villa E., Ebisawa M., Passalacqua G., Savi E., Ebo D., Gómez R.M. (2020). IgE allergy diagnostics and other relevant tests in allergy, a World Allergy Organization position paper. World Allergy Organ. J..

[128] Organisms E.P.o.G.M., Naegeli H., Birch A.N., Casacuberta J., De Schrijver A., Gralak M.A., Guerche P., Jones H., Manachini B., Messéan A. (2017). Guidance on allergenicity assessment of genetically modified plants. EFSA J..

